# Towards consistency in dietary pattern scoring: standardising scoring workflows for healthy dietary patterns using 24-h recall and two variations of a food frequency questionnair

**DOI:** 10.1017/S0007114524000072

**Published:** 2024-05-14

**Authors:** Lizanne Arnoldy, Sarah Gauci, Annie-Claude M. Lassemillante, Joris C. Verster, Helen Macpherson, Anne-Marie Minihane, Andrew Scholey, Andrew Pipingas, David J. White

**Affiliations:** 1 Centre for Mental Health and Brain Sciences, Swinburne University, Melbourne, VIC 3122, Australia; 2 IMPACT – the Institute for Mental and Physical Health and Clinical Translation, Food & Mood Centre, School of Medicine, Deakin University, Geelong, Australia; 3 Department of Nursing and Allied Health, Faculty of Health, Arts and Design, Swinburne University, Melbourne, VIC 3122, Australia; 4 Utrecht Institute for Pharmaceutical Sciences (UIPS), Division of Pharmacology, Utrecht University, 3584 CG Utrecht, The Netherlands; 5 Institute for Physical Activity and Nutrition (IPAN), School of Exercise and Nutrition Sciences, Deakin University, Geelong, VIC Australia; 6 Department of Nutrition and Preventive Medicine, Norwich Medical School, BCRE, University of East Anglia, Norwich, UK; 7 Nutrition Dietetics and Food, School of Clinical Sciences, Monash University, Melbourne, Australia

**Keywords:** Administered 24-H Dietary Assessment Tool, Dietary Approaches to Stop Hypertension, FFQ, Mediterranean-DASH Intervention for Neurodegenerative Delay

## Abstract

Healthy dietary patterns such as the Mediterranean diet (MeDi), Dietary Approaches to Stop Hypertension (DASH) and the Mediterranean-DASH Intervention for Neurodegenerative Delay (MIND) have been evaluated for their potential association with health outcomes. However, the lack of standardisation in scoring methodologies can hinder reproducibility and meaningful cross-study comparisons. Here we provide a reproducible workflow for generating the MeDi, DASH and MIND dietary pattern scores from frequently used dietary assessment tools including the 24-h recall tool and two variations of FFQ. Subjective aspects of the scoring process are highlighted and have led to a recommended reporting checklist. This checklist enables standardised reporting with sufficient detail to enhance the reproducibility and comparability of their outcomes. In addition to these aims, valuable insights in the strengths and limitations of each assessment tool for scoring the MeDi, DASH and MIND diet can be utilised by researchers and clinicians to determine which dietary assessment tool best meets their needs.

## Introduction

The role of diet and nutrition in health and well-being cannot be overstated. In recent years, research has shown that suboptimal diet and nutrition are major contributors to the global burden of non-communicable diseases^(1)^, resulting in 7·9 million deaths globally, and 187·7 million death and disability-adjusted life years in 2019^(1)^. Marked changes to how diet is conceptualised have occurred over the years with early research focused on the relationship between individual nutrients and health outcomes^(2)^. The recognition that nutrients do not function in isolation has led to research around whole dietary patterns, attempting to understand the complex interaction food components have on health outcomes. However, the methods used to characterise dietary patterns are inconsistent, adding to the challenge of generalising conclusions from individual studies and making meaningful between-study comparisons or translating findings into guidelines^(3)^.

Dietary patterns are described and quantified through various dietary assessment tools, which can be classified as ‘*a priori*’ and ‘*a posteriori*’ methods. Tools such as food frequency questionnaires (FFQs), 24-h diet recalls and food diaries are used to gather dietary data which are then analysed using ‘*a priori*’ methods. FFQ and 24-h diet recalls are more commonly used in epidemiological studies due to their reduced labour intensity compared with real-time recording methods such as food diaries^(4,5)^. Although all self-report methods have limitations, they are cost-efficient ways to gain insight into dietary habits. FFQ require individuals to report the frequency of food consumption and portion size, but provide little information about preparation methods or food combinations^(6)^. 24-h diet recalls, which can be self-reported or conducted through interviews and telephone calls, provide more detailed information by asking individuals to report all food and beverage consumption within the past 24 h^(7)^. In the current study, dietary patterns were determined using an *‘a priori*’ approach.

The computation of dietary pattern scores from *‘a priori*’ dietary assessment methods involves several subjective decisions, such as choosing assessment tools, dietary pattern scoring methods, food items or codes and determining the grams equivalent to a serving. Recent reviews by Wingrove et al. (2022) and Hutchins-Wiese et al. (2021) conclude that these decisions lead to considerable variability across the literature in the methods used, potentially affecting the strengths of associations. For example, the choice of assessment tool can cause differences in both the types and the number of food items included in a dietary pattern^(8)^. Furthermore, the degree of detail provided when describing these methods and dietary patterns is highly inconsistent, with most studies failing to describe the scored dietary patterns altogether^(3)^. Additionally, various studies use distinct scoring methods, as noted by Zaragoza-Martí et al. (2018), who reported that there are over twenty-eight unique scoring methods available for the Mediterranean diet (MeDi) score^(9)^. These variations in the scoring process currently hinder the synthesis of evidence into dietary guidelines or interventions^(10)^ and may contribute to the mixed and null effect sizes found in systematic reviews^(11–13)^. These findings highlight the need for standardisation and consensus on scoring systems and methodologies to capture dietary patterns. This will allow direct comparisons between studies and facilitate the translation of findings into dietary guidelines^(14)^.

Efforts have been made to standardise dietary pattern scores, such as the MeDi^(15)^, the Alternative Healthy Eating Index 2010^(16)^, Dietary Approaches to Stop Hypertension (DASH)^(17)^ and the Mediterranean-DASH Intervention for Neurodegenerative Delay (MIND) diet^(18)^. However, to our knowledge, no attempts have been made to standardise the entire process for scoring the MeDi, DASH or MIND, from dietary assessment tools to dietary patterns score. Many scientific disciplines are adopting practices to increase reproducibility, such as standardised methods and reporting guidelines^(19)^. A prominent example includes the Consolidated Standards of Reporting Trials statement, which has been universally adopted for randomised controlled trials^(20)^ with momentum gathering for the development of bespoke Consolidated Standards of Reporting Trials-Nutr guidance^(21)^. Similarly, the field of neuroimaging has worked towards reporting guidelines for measurement, data processing and statistical analysis elements that differ between studies and threaten reproducibility, via the Organisation for Human Brain Mapping established by the Committee on Best Practices in Data Analysis and Sharing^(22)^. In 2016, reporting guidelines on Strengthening the Reporting of Observational Studies in Epidemiology—Nutritional Epidemiology was published, providing a framework for reporting methods and results of associations with health outcomes^(23)^. Although there are no reporting recommendations specifically focussing on the MeDi, DASH and MIND scores, a related checklist published by Kirkpatrick et al. (2018) documents a reporting checklist for the Health Eating Index^(24)^, which can be used alongside the Strengthening the Reporting of Observational Studies in Epidemiology—Nutritional Epidemiology^(23)^.

To alleviate some of the bias and to contribute to efforts to further standardise this field, this study aimed to achieve two goals. First, it aimed to document a workflow that scores the MeDi, DASH and MIND dietary patterns from the outputs of three commonly used dietary assessment tools: one 24-h recall tool and two variations of an FFQ. This effort aims to further enhance reproducibility and enable better comparison across studies, ultimately enhancing our understanding of the association between dietary patterns and health outcomes. Second, it aimed to recommend a ‘best practice’ reporting checklist that highlights the essential elements identified in the dietary scoring workflow which require a description in a research paper to facilitate reproducibility and comparability of research studies, which builds on the checklist by Kirkpatrick et al. (2018). These recommendations may be of value to researchers, editors and reviewers, aiming to optimise the scoring practice and reporting in the field of nutritional research.

## Methods and materials

In order to achieve the stated objectives, secondary data representing common methods for assessing diet intake in research were identified. This led to the inclusion of baseline (pre-intervention cross-sectional data) assessments of three randomised controlled trials of nutritional interventions in middle-aged to older adults which were collected at Swinburne University, Melbourne, Australia. These trials incorporated different dietary assessment tools and were conducted in various countries with diverse populations. Scores for the MeDi, DASH and MIND dietary patterns were created through a systematic scoring process. Each stage of the process was thoroughly documented, with a specific focus on points where subjective decisions were made and potential variability could arise. Furthermore, the dietary pattern scores were evaluated against a set of variables known to be associated with the respective dietary patterns, aiming to validate their effectiveness.

### Data source

The three clinical trials included comprised the Memory and Attention Supplement Trial (MAST) NCT03482063^(25)^, the Phospholipid Intervention for Cognitive Ageing Reversal (PLICAR) trial ACTRN12613000347763^(26)^ and the Cognitive Ageing Nutrition and Neurogenesis (CANN) trial NCT02525198^(27,28)^. The MAST trial evaluated the impact of a 12-week intervention of vitamin B and herbal supplementation on cognition and mood in healthy middle-aged adults, while the PLICAR trial explored the neurocognitive effects of a 6-month supplementation with a phospholipid-rich milk protein (Lacprodan® PL-20, Arla Foods Ingredients, Denmark). The CANN trial was a dual-centre trial assessing the effect of a 12-month flavonoid/fatty acid supplementation on cognitive performance in individuals aged 55 years and over with mild cognitive impairment or subjective memory impairment. This study was conducted according to the guidelines laid down in the Declaration of Helsinki, and all procedures involving human patients were approved by the Swinburne University Human Research Ethics Committee (MAST: Project number 2017–269, PLICAR: project number 2012–294, CANN: Project number 2015-208) and the Bellberry Human Research Ethics Committee (CANN: Study ID 2015-03-227). In addition, ethical clearance from the Swinburne University Human Research Ethics Committee was received to utilise data from all three clinical trials for the present analysis (Project number 20202924–4284). Written informed consent was obtained from all participants.

### Participants

Eligibility criteria differed across the trials, ranging from healthy individuals free from any cognitive condition (MAST) or age-associated memory impairment (PLICAR), subjective memory impairment or mild cognitive impairment (CANN). Table 1 summarises in detail the inclusion and exclusion criteria of each study.

**Table 1.** A summary of the inclusion and exclusion criteria for each study: MAST, PLICAR and CANN

	MAST	PLICAR	CANN (UK site/Australia site)
Eligible participants (number of)	141	196	UK: 145 Australia:101
Age in years	40–65	55–75	55–85
Mean	53·3	65·5	UK: 65·7 Australia: 65·1
Sex (% Female)	50	54	UK: 52 Australia: 62
Cognitive function	Free from any cognitive or neurological conditions	Age-associated mild cognitive impairment	Mild cognitive impairment or subjective memory impairment
Inclusion	Free from cardiac diseases, psychiatric disorders including depression and anxiety, health conditions that could affect food absorption or events that could result in cognitive impairment	Fluent in English and free from a dementia diagnosis or any neurological, cardiac, endocrine, gastrointestinal or bleeding disorders	Fluent in English, had normal or corrected to normal vision and hearing, good general health and were free from an Alzheimer’s disease or dementia diagnosis, or condition or event that could result in cognitive impairment
Exclusion	Colour blindness, uncontrolled hypertension and the use of drugs, medication or supplements that could impact cognitive functioning	Smoker, a history of alcohol or substance abuse, use of cognitively affecting medications or substances, psychiatric illness, a rice allergy or a previous negative reaction to milk or dairy products	Uncontrolled hypertension, a diagnosis of gastrointestinal disorder, a history of alcohol or drug dependency, a high BMI, severe stenosis, a known allergy to fish or another component in the supplement or any other medical condition likely to affect the study measures
Dietary assessment tool	ASA24	CCV FFQ	EPIC FFQ

MAST, Memory and Attention Supplement Trial; PLICAR, Phospholipid Intervention for Cognitive Ageing Reversal; CANN, Cognitive Ageing Nutrition and Neurogenesis; UK, UK; ASA24, Administered 24-Hour Dietary Assessment Tool^(29)^; CCV, Cancer Council Victoria^(33)^; EPIC, European Prospective Investigation into Cancer and Nutrition^(35)^.

### Diet

#### Dietary assessments

Each of the three clinical trials used a different dietary assessment tool to estimate participants’ dietary food habits and nutrient intake, as presented in Table 1 and outlined in the following sections.

##### Administered 24-H Dietary Assessment Tool

The MAST trial utilised the Administered 24-H Dietary Assessment Tool (ASA24) tool that collects information on the dietary intake of participants over 24 h^(29)^. The ASA24 is a web-based dietary assessment tool developed by the National Cancer Institute that has been validated and compared with other established dietary assessment methods^(30,31)^. Participants were required to report all the foods, drinks and supplements consumed in the specified timeframe and answer questions regarding preparation, food form, portion size and meal additions^(29)^. A total of four diet recalls were completed by participants during the trial, with the first completed during an on-site testing session under researcher supervision. Participants completed the second dietary assessment before their baseline session, with one completed during the week and one during the weekend. Two additional recalls were completed in the week leading up to the endpoint testing session. Information collected from a minimum of two and a maximum of four recalls was used to calculate adherence to the MeDi, DASH and MIND dietary pattern, thereby providing a more representative capture of participants’ habitual diets^(32)^.

The ASA24 assessment tool incorporates the Australian Food, Supplement, and Nutrient Database (AUSNUT 2011–2013) as well as the 2011–2013 Australian Health Survey (AHS) as its guiding reference. The raw data generated from ASA24 comprises food consumption in grams, and each food is coded using the AUSNUT 2011–2013 food-nutrient database.

AUSNUT 2011–2013 covers a wide range of food and drink components, comprising a total of 5740 items. This food-nutrient database comprises eleven files of which three were used for data analysis: the food recipe file which was used to disaggregate mixed dishes; the food and dietary supplement classification system file and the food details file. The classification system file uses codes for major (two-digit), sub-major (three-digit), minor (five-digit) and survey ID (eight-digit) category codes to classify components of the different categories of dietary patterns. To give an example, the two-digit codes classified whole food groups, the three-digit codes classified specific components of a food group, five-digit codes were used to classify green leafy vegetables in the vegetable component, while eight-digit codes were needed to disaggregate mixed dishes and to classify more detailed codes such as separating whole grains from total grain intake. The classification system file and the food details files were used to classify AUSNUT 2011–2013 food codes based on their alignment with the foods in the MeDi, DASH and MIND dietary patterns. To accomplish this, we extracted all the codes and incorporated them into their corresponding components, as outlined in Tables 2, 3, 4.

**Table 2.** Includes the food items extracted from the ASA24 (which also includes the AUSNUT codes), CCV FFQ and EPIC FFQ for the MeDi dietary pattern

MeDi Questions	ASA24		CCV FFQ	EPIC FFQ
	Included items (serving size in gram)	AUSNUT codes	Included items (serving size in grams)	Included items (serving size in grams)
1. Do you use olive oil as main culinary fat?	Olive oil, plant oils (%)	14402007, 14	NA	NA
2. How much olive oil do you consume in a given day (including oil used for frying, salads, out of house meals, etc?)	Olive oil (13·5)	14402007	NA	NA
3. How many vegetable servings do you consume per day?	Bok choy, brussels sprouts, cabbage, kale, kohlrabi, broccoli, cauliflower, carrot, artichoke, beetroot, cassava, celeriac, chicory, ginger, parsnip, radish, swede, taro, turnip, wasabi, endive, lettice, mixed leafy greens, rocket, silver beet, spinach, vine leaf, watercress, asparagus, bamboo, celery, basil, chives, coriander, dill, flower, herbs, mint, parsley, rosemary, tomato, pumpkin, squash, zucchini, mushroom, sweetcorn, avocado, capsicum, chilli, choko, cucumber, eggplant, melon, okra, fennel, garlic, leek, onion, potatoes, wild harvested vegetables, vegetable juice, fruit/vegetable juice blends, vegetable- based pickles (gherkin, ginger, olive, pickles, relish), vegetable dips (200)	11305, 11306, 23202, 23502, 24001, 24101, 242-244, 246-248	Avocado, potatoes (not fat), tomato sauce, tomatoes, capsicum, lettuce, cucumber, celery, beetroot, carrots, cabbage, cauliflower, broccoli, spinach, peas, bean sprouts, pumpkin, onion, garlic, mushrooms, zucchini (200)	Avocado, beansprouts, beetroot, broccoli, broccoli salted, broccoli unsalted, cabbage, cabbage salted, cabbage unsalted, carrots, carrots salted, carrots unsalted, cauliflower, cauliflower salted, cauliflower unsalted, coleslaw, garlic, leeks, leeks salted, leeks unsalted, marrow, marrow salted, marrow unsalted, mushrooms, olive spread, onions, parsnips, parsnips salted, parsnips unsalted, peas, peas salted, peas unsalted, peppers, pickles, potato salad, potatoes, potatoes salted, potatoes unsalted, roast potatoes only, salad, spinach, spinach salted, spinach unsalted, sprouts, sprouts salted, sprouts unsalted, squash, sweetcorn, tomatoes, vegetable soup, watercress (200)
4. How many fruit units (including natural fruit juices) do you consume per day?	Wild harvested fruits, apple, pear, loquat, quince, blackberry, cranberry, mulberry, raspberry, strawberry, orange, lemon, lime, cumquat, grapefruit, mandarin, tangelo, tangerine, mandarin, nectarine, peach, apricot, cherry, plum, banana, pineapple, babaco, cheese fruit, fig, persimmon, tamarillo, wax jambul, feijoa, guava, jackfruit, lychee, mango, passionfruit, pawpaw, pomegranate, prickly pear, rambutan, grape, kiwifruit, melon, pepino, rhubarb, quandong, currant, raisin, sultana, fruit juices (freshly squeezed) (150)	11302, 16	Tinned fruit, oranges, apples, pears, bananas, melon, pineapple, strawberries, apricots, peaches, mango (150)	Apples, bananas, dried, fruit juice, grapefruit, grapes, melon, oranges, peaches, pears, strawberries, tinned (150)
5. How many servings of red meat, hamburger or meat products (ham, sausage, etc.) do you consume per day?	Unprocessed beef, lamb, mutton, pork, veal, kangaroo, buffalo, camel, goat, rabbit, venison, sausages, frankfurt’s and saveloys, bacon, ham, prosciutto, kabana, salami, chorizo, mortadella, jerky, berliner, devon, wild harvested mammalian meat (100)	18011, 181, 182, 185, 186	Beef, veal, lamb, pork, bacon, ham, salami, sausages, hamburger (100)	Bacon fat, bacon lean, beef fat, beef lean, ham, lamb fat, lamb lean, pork fat, pork lean, sausages, spam, burgers (100)
6. How many servings of butter, margarine, or cream do you consume per day?	Butters, margarine and table spreads, dairy blends, unspecified dairy-based fat or margarine used as a spread, ghee, cream, dairy-based savoury sauces (12)	141-143, 14601, 193, 23108	Full cream milk, margarine, polyunsaturated margarine, monounsaturated margarine, butter and margarine blends, butter (12)	Block margarine, butter, double cream, other margarine, polyunsaturated margarine, RF butter, single cream, FF salad cream, low-fat salad cream, low-fat spread, very low-fat spread (12)
7. How many sweet/carbonated beverages do you drink per day?	Fruit juice/ drink, cordial, soft drink, sweetened caffeinated colas, sugar-sweetened caffeine-free colas, other sugar-sweetened carbonated drinks, lemonade or other noncarbonated fruit drink, electrolyte drinks, energy drinks (times/day)	11302, 11307, 11309, 114-116, 118	Fruit juice (times/day)	Fizzy drinks, fruit juice, LC drinks (times/day)
8. How much wine do you drink per week?	Red and white wine (including sparkling varieties and rose styles), fortified wines, reduced alcohol wines (150 ml)	29201-29204	Red wine, white wine, fortifies wine (150 ml)	Port, red wine, white wine, wine red and white (150 ml)
9. How many servings of legumes do you consume per week?	Broad bean, butter bean, green bean, red bean, black bean, haricot bean, lima bean, lupin bean, red kidney bean, soybean, cannellini bean, baked beans, sprouts, chickpea, lentil, pea, snow pea, tempeh, tofu, legume and pulse products, legume-based dips (hummus), lentil or other legumes soup (150)	20601006, 23503, 245, 251, 25201	Green beans, baked beans, tofu, other beans (150)	Baked beans, beans, beans salted, beans unsalted, pulses (150)
10. How many servings of fish or shellfish do you consume per week?	Fin fish (barramundi, bassa, blue grenadier, blue-eye trevalla, bream, cod, flathead, flounder, gemfish, grouper, john dory, ling, mackerel, milkfish, moronga, mullet, mulloway, nile perch, orange, salmon, sardine, shark, silver perch, snapper, swordfish, tilapia, kingfish, trout, tuna, whitebait, whiting, caviar, eel, anchovy, herring) (125), wild caught fish and seafood (125), crab (200), lobster (200), moreton bay bug (200), prawn (200), mussel (200), octopus, oyster (200), scallop (200), squid or calamari (200)	151-154, 15501001, 15501003, 15501004, 15501008, 15501011, 15501014-15501016, 15501019, 15501020, 15501022-15501024, 15501027, 15501028, 15501030-15501033, 15501035, 15501036, 15501038, 15502001-15502003, 157	Fish (125), Tinned fish (125), Fried fish (125)	Fish fingers (125), fish roe (125), fried fish (125), oily fish (125), white fish (125), shellfish (200)
11. How many times per week do you consume commercial sweets or pastries (not homemade), such as cakes, cookies, biscuits, or custard?	Sweet bread, buns and scrolls, sweet biscuits, cakes, muffins, scones, waffles, doughnut, crumpets, cake-type desserts, plain and sweet pastry, pancakes, crepes, ice cream, gelato, custards, dairy desserts, rice pudding, cheesecake, trifle, tiramisu, jelly, pavlova, meringue, chocolate, confectionery (times/week)	12305, 12306, 131, 133, 13401-13403, 136, 196, 273, 281, 28202, 284	Jam, honey, ice-cream, chocolate, flavoured milk, sweet biscuits, cakes (times/week)	Buns/pastries home baked, buns/pastries ready-made, cakes home baked, cakes ready-made, low cholesterol chocolate biscuits, chocolate milk and dark, low cholesterol spreads, dairy desserts, dark chocolates, frosties, fruit pies home baked, fruit pies ready-made, ice cream, jam, low fat hot choc, milk chocolates, milk puddings, puddings home baked, puddings ready-made, sugar, sweets (times/week)
12. How many servings of nuts (including peanuts) do you consume per week?	Nuts and seeds (30)	22, 16803	Nuts (30)	Salted nuts, unsalted nuts, peanut butter, nuts salted and unsalted, seeds (30)
13. Do you preferentially consume chicken, turkey or rabbit meat instead of veal, pork, hamburger or sausage?	Chicken, duck, turkey, emu, mutton-bird, ostrich, pigeon, quail (%)	183	Chicken, beef, veal, chicken, lamb, pork, bacon, ham, salami, sausages	Bacon fat, bacon lean, beef fat, beef lean, ham, lamb fat, lamb lean, pork fat, pork lean, sausages, spam, burgers, chicken
14. How many times per week do you consume vegetables, pasta, rice, or other dishes seasoned with sofrito?	Tomato-based products and dishes and tomato-based sauces (not tomato sauce aka ketchup) (Times/week)	23104, 23106	Tomato sauce (times/week)	NA
Number of components included in score		14	12	11

ASA24, Automated Self-Administered 24-Hour Dietary Assessment Tool; CCV, Cancer Council; EPIC, European Prospective Investigation into Cancer and Nutrition; Medi, Mediterranean diet.

This table presents the included items from the ASA24, CCV FFQ and EPIC FFQ in the MeDi and presents the grams equivalent to a serving size of each item utilised for scoring the MeDi, indicated in brackets.

**Table 3.** Includes the food items extracted from the ASA24 (which also includes the AUSNUT codes), CCV FFQ and EPIC FFQ for the DASH dietary pattern

	ASA24		CCV FFQ	EPIC FFQ
DASH components	Included items (serving size in grams)	AUSNUT codes	Included items (serving size in grams)	Included items (serving size in grams)
Total grain intake	Grains for bread (43), cooked barley (100), buckwheat groats (100), bulgur (100), cornmeal cooked (100) uncooked (57), oats (uncooked) (57), quinoa (100), rice (100), rye cooked (100) uncooked (57), spelt uncooked (57), couscous cooked (100) uncooked (57), flour (semolina, cornflour, rye, spelt, wholemeal) (57), tapioca, flatbread (43), muffins (43), noodles cooked (100), pasta (100), breakfast cereal (26), muesli (57), porridge (110), Cakes (57), scones (57), cake-type desserts (57), savoury biscuits (57), pancakes (57), crepes (57), waffles (57), popcorn (57), muesli or cereal bars (57), crumpets (57)	12, 12514, 12515, 132, 133, 13601–13603, 13606, 26202, 283	High fibre white bread (43), white bread (43), wholemeal bread (43), rye bread (43), multigrain bread (43), all bran (26), brand flakes (26), weet bix (26), cornflakes (26), porridge (110), muesli (57), rice (100), pasta (100), crackers (57), sweet biscuits (57), cakes (57)	Breakfast cereal general (26), brown bread (43), brown rice (100), buns HB (43), buns RM (43), cakes HB (57), cakes RM (57), cereal (26), cereal bars (57), chocolate biscuits (57), crackers (57), crispbread (57), crisps (57), frosties (26), HF cereals (26), horlicks (57), muesli (57), naan (43), plain biscuits (57), porridge (110), RF biscuits (57), white bread (43), white pasta (100), white rice (100), wholemeal bread (43), wholemeal pasta (100)
Whole grain intake	Whole grain bread (43), muffin (57), noodles cooked (100), pasta (100), brown/red rice (100), whole grain cold breakfast cereal (26), hot porridge (110), savoury biscuits (57), muesli and cereal-style bars (57), cooked barley (100), bulgur (100), cornmeal cooked (100), millet, oats (uncooked) (57), quinoa cooked (100), couscous cooked (100) uncooked (57), tapioca, whole grain flour (57), popcorn (57)	12101001–12101003, 12101006–12101011, 12101014–12101022, 12101025, 12101026, 12101030, 12102007–12102009, 12102014, 12103001–12103003, 12103006, 12103007, 12103010, 12103011, 12103014, 12103015, 12103021, 12103022, 12201009, 12201010, 12203013, 12203014, 12204–12212, 12214001, 12301004, 12301005, 12302004, 12302005, 12302008–12302011, 12303, 12401018–12401020, 12402, 12403002, 12403007, 12502, 12505, 12506, 12511, 12512, 12514, 12515, 12516001, 13201001, 13201002, 13201008–13201012, 13203, 13204001–13204003, 13205001, 13205002, 12403006, 12501, 12507, 12513, 126, 26202, 28301	Wholemeal bread (43), rye bread (43), multigrain bread (43), brand flakes (26), weet bix (26), porridge (100), muesli (57)	Brown bread (43), brown rice (100), cereal (26), cereal bars (57), crispbread (43), HF cereals (26), muesli (57), porridge (110), wholemeal bread (43), wholemeal pasta (100)
Vegetables	Endivev (35), lettuce (35), mixed leafy greens (35), rocket (35), silver beet (35), spinach (35), nine leaf (35), watercress (35), Bok choy (35), brussels sprout (35), cabbage (35), kale (35), kohlrabi (35), potatoes (70), tomato and tomato products (70), pumpkin (70), squash and zucchini (70), mushrooms (70), sweetcorn (70), avocado (70), capsicum (70), chilli (70), choke (70), cucumber (70), eggplant (70), melon (70), okra (70), fennel (70), water chestnut (70), garlic (70), leek (70), onion (70), shallot (70), broccoli (70), broccolini (70), cauliflower (70), wild harvested vegetables (70), peas (70), sprouts (70), carrot (70), beetroot (70), cassava (70), celeriac (70), ginger (70), radish (70), chicory (70), swede (70), taro (70), turnip (70), wasabi (70), artichoke (70), asparagus (70), bamboo shoot (70), celery (70), wild harvested (70), green/ snow peas (70), alfalfa sprouts (70), bean sprouts (70), freshly squeezed vegetable juices (120), fruit and vegetable juice blends (120), vegetable-based pickles (70), chutneys (70), relishes and dips (70)	11305, 11306, 23104, 23106, 202, 23502, 24001, 24101, 242–244, 246–248, 24501, 24503,	Avocado (70), potatoes (70), tomato sauce (70), tomatoes (70), capsicum (70), lettuce (35), cucumber (70), celery (70), beetroot (70), carrots (70), cabbage (35), cauliflower (70), broccoli (70), spinach (35), peas (70), bean sprouts (70), pumpkin (70), onion (70), garlic (70), mushrooms (70), zucchini (70)	Avocado (70), beansprouts (35), beetroot (70), broccoli (35), broccoli salted (35), broccoli unsalted (35), cabbage (35), cabbage salted (35), cabbage unsalted (35), carrots (70), carrots salted (70), carrots unsalted (70), cauliflower (35), cauliflower salted (35), cauliflower unsalted (35), coleslaw (35), garlic, leeks (35), leeks salted (35), leeks unsalted (35), marrow (70), marrow salted, marrow unsalted (70), mushrooms (70), olive spread (120), onions (70), parsnips (35), parsnips salted (35), parsnips unsalted (35), peas, peas salted (70), peas unsalted (70), peppers (70), pickles (70), potato salad (70), potatoes (70), potatoes salted (70), potatoes unsalted (70), roast potatoes only (70), salad (35), spinach (35), spinach salted (35), spinach unsalted (35), sprouts, sprouts salted (70), sprouts unsalted (70), squash (70), sweetcorn (70), tomatoes (70), vegetable soup (120), watercress (35)
Fruits	Fruits (wild harvested fruits, apple, pear, loquat, quince, berries, orange, lemon, lime, cumquat, grapefruit, mandarin, tangelo, tangerine, mandarin, nectarine, peach, spricot, cherry, plum, banana, pineapple, babaco, cheese fruit, fig, persimmon, tamarillo, wax jambul, feijoa, guava, jackfruit, lychee, mango, passionfruit, pawpaw, pomegranate, prickly pear, rambutan, grape, kiwifruit, melon, pepino, rhubarb, plum, quandong, currant, raisin, sultana) (150*), fruit juices (freshly squeezed) (124)	16, 11302	Tinned fruit (113), fruit juice (124), oranges (150), apples (182), pears (150), bananas (118), melon (150), pineapple (150), strawberries (67), apricots (150), peaches (150), mango (150)	Apples (182), bananas (118), dried (28), fruit juice (124), grapefruit (150), grapes (150), melon (150), oranges (150), peaches (150), pears (150), strawberries (67), tinned fruit (113)
Dairy	Cow and goat milk (246), yoghurt (246), cream (214*), blue vein cheese (42·5), cheddar (42·5), cheshire (42·5), colby style (42·5), edam (42·5), fetta (42·5), goat cheese (42·5), Gloucester style (42·5), gouda, (42·5) haloumi (42·5), Havarti style (42·5), Jarlsberg (42·5), mozzarella (42·5), parmesan (42·5), pecorino (42·5), provolone (42·5), romano (42·5), swiss (42·5), bocconcini (42·5), cottage (42·5), cream cheese (42·5), Neufchatel (42·5), ricotta (42·5), brie (42·5), camembert (42·5), ice cream (246), frozen yoghurt (246), sundae, custard (246), dairy dessert (246), formia’s frais (246), pudding (246), cheesecake (246), trifle (246), tiramisu (246), iced coffee (246), milkshake (246), thick shake (246), milk-based fruit drinks (246), dairy-based savoury sauces (214*), dairy-based dips (214*)	181, 182, 185, 186, 18011	Full cream milk (246), reduced fat milk (246), skim milk (246), hard cheese (42·5), firm cheese (42·5), soft cheese (42·5), ricotta or cottage cheese (42·5), cream cheese (42·5), low-fat cheese (42·5), flavoured milk drinks (246), ice cream (148), yoghurt (246)	Cheese (42·5), cottage cheese (42·5), dairy desserts (246), double cream (214), FF salad cream (214), FF yoghurt (246), horlicks (246), ice cream (246), LF cheese (42·5), LF hot choc (246), LF salad cream (214), LF yoghurt (246), milk chocolates (246), milk dried (246), milk full (246), milk goat (246), milk one percent (246), milk other full (246), milk other semi (246), milk semi (246), milk skimmed (246), a milk soya (246), single cream (214)
Meats, poultry, and fish	Unprocessed beef (85), lamb (85), mutton (85), pork (85), veal (85), kangaroo (85), buffalo (85), camel (85), goat (85), rabbit (85), venison (85), sausages (85), Frankfurt’s and saveloys (85), bacon (85), ham (85), prosciutto (85), kabana (85), salami (85), chorizo (85), mortadella (85), jerky (85), wild harvested mammalian meat (85), chicken (85), duck (85), turkey (85), emu (85), mutton-bird (85), ostrich (85), pigeon (85), quail (85), Barramundi (85), bass (85), blue grenadier (85), blue-eye trevalla (85), bream (85), cod (85), flathead (85), flounder (85), gemfish (85), grouper (85), John Dory (85), ling (85), mackerel (85), milkfish (85), moronga (85), mullet (85), mulloway (85), Nile perch (85), orange (85), salmon (85), sardine (85), shark (85), silver perch (85), snapper (85), swordfish (85), tilapia (85), kingfish (85), trout (85), tuna (85), whitebait (85), whiting (85), caviar (85), eel (85), anchovy (85), herring (85), wild caught fish and seafood (85), crab (85), lobster (85), Moreton bay bug (85), prawn (85), Mussel (85), octopus (85), oyster (85), scallop (85), squid or calamari (85), egg (50), wild harvested eggs (50), savoury egg dishes (50)	151, 153, 154, 157	Beef (85), veal (85), chicken (85), lamb (85), pork (85), bacon (85), ham (85), salami (85), sausages (85), fish (85), fried fish (85), tinned fish (85), eggs (50)	Bacon fat, bacon lean, beef fat, beef lean, burgers, chicken, fish fingers, fish roe, fried fish, ham, lamb fat, lamb lean, oily fish, pork fat, pork lean, sausages, shellfish, spam, white fish (85), eggs (50)
Nuts, seeds and dry beans	Dried fruit and nut mixes (42·5), tempeh (98), tofu (98), lentil/ legumes soup (98), seeds (chi, linseed, flaxseed, poopy, pumpkin, sesame, sunflower, acacia, psyllium, tahini) (42·5), nuts (peanuts and products, coconut, almond, cashew chestnut hazelnut, macadamia, pecan, pine, pistachio, walnut, pandanus, Brazil) (42·5), wild harvested seeds and nuts (42·5), hummus (98), beans (black, haricot, lima, lupin, red kidney, soya, cannellini, lupin) (98), chickpea (98), lentil (98), pea (98), legume and pulse products (miso, chick pea/ soya flour, baked beans) (98)	183, 18903001, 18903002, 18903005–18903011, 18903014–18903023, 18903048–18903054	Nuts, peanut butter (42·5)	Baked beans (98), beans (98), beans salted (98), beans unsalted (98), cocoa (42·5), peanut butter (42·5), nuts salted and unsalted (42·5), pulses (98), salted nuts (42·5), unsalted nuts (42·5), seeds (42·5)
% kcal from fat	Total energy, fat	20601006–20601012, 21602001, 23503, 24502, 251, 25201	Total energy, fat	Total energy, fat
% kcal from saturated fatty acids	Total energy, saturated fat	22, 16803	Total energy, saturated fat	Total energy, saturated fat
Sweets	Sweetened caffeinated colas (372), sugar-sweetened caffeine-free colas (372), other sugar-sweetened carbonated drinks (372), lemonade or other noncarbonated fruit drink (372), electrolyte drinks (372), energy drinks (372), sweet bread (100), buns and scrolls (100), sweet biscuits (100), cakes (100), muffins (100), scones (100), cake-type desserts (100), plain and sweet pastry (100), pancakes (100), crepes (100), waffles (100), doughnut (100), Crumpets (100), ice cream, Gelato (119), Custards (100), dairy desserts (100), rice pudding (100), cheesecake (100), trifle (100), tiramisu (100), milkshake (100), thick shake, iced coffee (119), iced chocolate, sugar (18), honey and sugar syrups (18), toppings (18), icing (18), sweet spreads (18), Jelly (18), pavlova (100), meringue (100), chocolate (100),	12307, 15502004, 24102, 15501002, 15501006, 15501007, 15501010, 15501012, 15501013, 15501017, 15501018, 15501021, 15501026, 15501029, 15501034, 15501037, 15501039, 15501040, 18903025–18903047, 18903012, 18903013, 13501, 13502, 13503037–13503052, 13505–13508, 136, 26	Sugars (18), jam (18), ice cream (100), chocolate (100), flavoured milk drinks (100), sweet biscuits (100), cakes (100)	Buns/pastries home baked (100), buns /pastries ready-made (100), cakes home baked (100), cakes ready-made (100), chocolate biscuits (100), chocolate milk and dark, cholesterol low spreads (100), Dairy desserts (100), Dark chocolate (100)s, frosties (100), fruit pies home baked (100), fruit pies ready-made (100), ice cream (100), Jam (18), low C drinks (372), low-fat hot choc (100), milk chocolates (100), milk puddings (100), puddings home baked (100), puddings ready-made (100), sugar (18), sweets (100)
Sodium	Na (mg/day)	14402007	Na (mg/day)	Na (mg/day)
Number of components included in score		11	11	11

ASA24, Automated Self-Administered 24-Hour Dietary Assessment Tool; CCV, Cancer Council; DASH, Dietary Approaches to Stop Hypertension; EPIC, European Prospective Investigation into Cancer and Nutrition.

This table presents the included items from the ASA24, CCV FFQ and EPIC FFQ in the DASH diet and presents the grams equivalent to a serving size of each item utilised for scoring the DASH, indicated in brackets.

* Average.

**Table 4.** Includes the food items extracted from the ASA24 (which also includes the AUSNUT codes), CCV FFQ and EPIC FFQ for the MIND

	ASA24		CCV FFQ	EPIC FFQ
MIND components	Included items (serving size in grams)	AUSNUT codes	Included items (serving size in grams)	Included items (serving size in grams)
Whole grains	Whole grain bread (43), muesli (38), noodles cooked (133) uncooked (38), pasta cooked (133) uncooked (38), brown/red rice cooked (133) uncooked (38), whole grain cold breakfast cereal (38), hot porridge (180), savoury biscuits (57), muesli and cereal style bars (57), barley cooked (133) uncooked (38), bulgur cooked (133) uncooked (38), cornmeal cooked (133), millet cooked (133), oats uncooked (57), quinoa cooked (133) uncooked (38), couscous cooked (133) uncooked (38), tapioca (133), whole grain flour uncooked (57), popcorn (57)	12101001–12101003, 12101006–12101011, 12101014–12101022, 12101025, 12101026, 12101030, 12102007–12102009, 12102014, 12103001–12103003, 12103006, 12103007, 12103010, 12103011, 12103014, 12103015, 12103021, 12103022, 12201009, 12201010, 12203013, 12203014, 12204-12212, 12214001, 12301004, 12301005, 12302004, 12302005, 12302008-12302010, 12302011, 12303, 12401018–12401020, 12402, 12403002, 12403007, 12516001, 12502, 12505, 12506, 12511, 12512, 12514, 12515, 126, 13201001, 13201002, 13201008–13201012, 13203, 13204001, 13204002, 13204003, 13205001, 13205002, 12403006, 12501, 12507, 12513, 26202, 28301	Wholemeal bread (43), rye bread (43), multi grain bread (43), brand flakes (38), weet bix (38), porridge cooked (180), muesli (38)	Brown bread (43), brown rice cooked (133), cereal (38), cereal bars (57), crispbread (57), high-fibre cereals (38), muesli (38), porridge (180), wholemeal bread (43), wholemeal pasta (133)
Green leafy vegetables	Endive, lettuce, mixed leafy greens, rocket, silver beet, spinach, nine leaf, watercress, bock choy, brussels sprout, cabbage, kale, kohlrabi (35)	24401, 24201	Lettuce, cabbage, spinach (35)	Cabbage, cabbage salted, cabbage unsalted, Coleslaw, spinach, spinach salted, spinach unsalted, watercress (35)
Other vegetables	Vegetables (potatoes, tomato and tomato products, pumpkin, squash and zucchini, mushrooms, sweetcorn, avocado, capsicum, chilli, choke, cucumber, eggplant, melon, okra, fennel, water chestnut, garlic, leek, onion, broccoli, broccolini, cauliflower, wild harvested vegetables, peas, sprouts, carrot, beetroot, cassava, celeriac, ginger, radish, chicory, artichoke, asparagus, bamboo shoot, celery) (70), seaweeds (70), vegetable-based (pickles, chutneys, relishes and dips) (70), freshly squeezed vegetable juices (120)	11305, 23104, 23106, 23202, 23502, 24001, 24101, 24202, 243, 24402, 24404, 24501, 24503, 246, 247, 248	Avocado, potatoes, tomato sauce, tomatoes, capsicum, cucumber, celery, beetroot, carrots, cauliflower, broccoli, peas, bean sprouts, pumpkin, onion, garlic, mushrooms, zucchini (70)	Avocado, beansprouts, beetroot, broccoli, broccoli salted, broccoli unsalted, carrots, carrots salted, carrots unsalted, cauliflower, cauliflower salted, cauliflower unsalted, garlic, leeks, leeks salted, leeks unsalted, marrow, marrow salted, marrow unsalted, mushrooms, olive spread, onions, parsnips, parsnips salted, parsnips unsalted, peas, peas salted, peas unsalted, peppers, pickles, potato salad, potatoes, potatoes salted, potatoes unsalted, roast potatoes only, salad, sprouts, sprouts salted, sprouts unsalted, squash, sweetcorn, tomatoes (70), vegetable soup (120)
Berries	Blackberry, cranberry, mulberry, raspberry, strawberry, goji berry (67)	162, 16802005, 16802008, 16802009	Strawberries (67)	Strawberries (67)
Red meat + products	Unprocessed beef, lamb, mutton, pork, veal, kangaroo, buffalo, camel, goat, rabbit, venison, sausages, Frankfurt’s and saveloys, bacon, ham, prosciutto, kabana, salami, chorizo, mortadella, jerky, wild harvested mammalian meat (85)	181, 182, 185, 186, 18011	Beef, veal, lamb, pork, bacon, ham, salami, sausages (85)	Bacon fat, bacon lean, beef fat, beef lean, ham, lamb fat, lamb lean, pork fat, pork lean, sausages, spam (85)
Fish	Fin fish (barramundi, bassa, blue grenadier, blue-eye trevalla, bream, cod, flathead, flounder, gemfish, grouper, John Dory, ling, mackerel, milkfish, moronga, mullet, mulloway, Nile perch, orange, salmon, sardine, shark, silver perch, snapper, swordfish, tilapia, kingfish, trout, tuna, whitebait, whiting, caviar, eel, anchovy, herring, wild caught fish and seafood (85)	151, 153, 154, 157	Fish, tinned fish (85)	Fish roe, oily fish, shellfish, white fish (85)
Poultry	Chicken, Duck, turkey, emu, Mutton-bird, Ostrich, Pigeon, Quail (85)	183, 18903001, 18903002, 18903005–18903011, 18903014–18903023, 18903048–18903054	Chicken (85)	Chicken (85)
Beans	Bean, black bean, haricot bean, lima bean, lupin bean, red kidney bean, soybean, cannellini bean, baked beans, chickpea, lentil, pea, tempeh, tofu, legume-based dips, lentil or other legumes soup (98)	20601006–20601012, 21602001, 23503, 24502, 251, 25201	Green beans (98), baked beans (98), tofu (98), other beans (98)	Baked beans, beans, beans salted, beans unsalted, pulses (98)
Nuts	Nuts and seeds (42·5)	22, 16803	Nuts (42·5)	Salted nuts, unsalted nuts, peanut butter, nuts salted and unsalted, seeds (42·5)
Fast/fried foods	How often do you eat fried food away from home (like French fries, chicken nuggets (times/day)	12307, 15502004, 24102, 15501002, 15501006, 15501007, 15501010, 15501012, 15501013, 15501017, 15501018, 15501021, 15501026, 15501029, 15501034, 15501037, 15501039, 15501040, 18903025–18903047, 18903012, 18903013, 13501, 13502, 13503037–13503052, 13505–13508, 136, 26	Fried fish, meat pies, pizza, hamburger (times/day)	Burgers, chips, chips only, fish fingers, fried fish, pizza (times/day)
Olive oil	Olive oil consumption was scored 1 if identiﬁed by the participant as the primary oil usually used at home and 0 otherwise (> = 50 % of average intake: (gr olive oil/gr total fat × 100))	14402007	NA	NA
Butter, margarine	Butters, margarine and table spreads, dairy blends, unspecified dairy-based fat or margarine used as a spread (14)	141–142, 14601	Margarine, polyunsaturated margarine, monounsaturated margarine, butter and margarine blends, butter (14)	Block margarine, butter, LF spread, other margarine, polyunsaturated margarine, RF butter, very-low-fat spread (14)
Cheese	Blue vein cheese, cheddar, Cheshire, Colby style, edam, fetta, goat, Gloucester style, Gouda, Haloumi, Havarti style, Jarlsberg, Mozzarella, Parmesan, Pecorino, provolone, Romano, Swiss, bocconcini, cottage, cream cheese, ricotta, brie, camembert (42·5)	194	Hard cheese, firm cheese, soft cheese, ricotta or cottage cheese, cream cheese, low-fat cheese (42·5)	Cheese, cottage cheese, low-fat cheese (42·5)
Pastries, sweets	Sugar (18), honey and sugar syrups (18), toppings (18), jam (18), marmalade (18), sweet spreads or sauces (18), jelly (18), meringue (18), pavlova (100), sorbet (119), gelato (119), icing (18), chocolate/ chocolate bar (100), sweet biscuits (100), cakes (100), muffins (100), scones(100), cake-type desserts (100), brownie (100), crepe (100), pancake (100), pikelet (100), waffle (100), fritter (100), doughnuts (100), crumpets (100), ice cream (199), frozen yoghurt (199), dairy desserts (100), flavoured milks/milkshakes (152), caramels (18), fudge (18), liquorice (18), lolly (18), lollipop (18), marshmallow (18), sherbet (18), Turkish delight (18), chewing gum (18), sweet breads (100), sweet biscuits (100), pastry (100), nut and seed-based confectionery (18)	12305, 12306, 131, 133, 13401–13403, 136, 195–198, 281, 284, 27, 28202	Sugars (18), jam (18), ice cream (119), chocolate (100), flavoured milk drinks (152), sweet biscuits (100), cakes (100)	Buns/pastries home baked (100), buns /pastries ready-made (100), cakes home baked (100), cakes ready-made (100), chocolate biscuits (100), chocolate milk and dark (152), cholesterol low spreads (100), dairy desserts (100), dark chocolates (100), frosties (100), fruit pies home baked (100), fruit pies ready-made (100), ice cream (119), jam (100), low fat hot choc (152), milk chocolates (100), milk puddings (100), puddings home baked (100), puddings ready-made (100), sugar, sweets (100)
Wine	Red and white wine (including sparkling varieties and rose styles), fortified wines, reduced alcohol wines (141·7)	29201–29204	Red wine, white wine, fortifies wine (141·7)	Port, red wine, white wine, wine red and white (141,7)
Number of components included in score		15	14	14

ASA24, Automated Self-Administered 24-Hour Dietary Assessment Tool; CCV, Cancer Council; EPIC, European Prospective Investigation into Cancer and Nutrition; MIND, Mediterranean-DASH Intervention for Neurodegenerative Delay.

This table presents the included items from the ASA24, CCV FFQ and EPIC FFQ in the MIND and presents the grams equivalent to a serving size of each item utilised for scoring the MIND, indicated in brackets.

To disaggregate reported dishes into their separate ingredients, the AUSNUT 2011–2013 recipe file was used as it includes information on the percentage of each ingredient included in each dish and the total weight. After which, the weights of each ingredient were calculated. For dishes not included in the AUSNUT 2011–2013 recipe file, efforts were made to find similar dishes. If no match was found, the dish was excluded from the analysis. Next, the ingredients that are part of the MeDi, DASH and MIND dietary patterns were extracted from the disaggregated data. The participants reported a total of 283 dishes, which were disaggregated for analysis. Details on these dishes are listed in online Supplementary Material A Table S1.

##### Cancer Council Victoria FFQ

The PLICAR trial used the Cancer Council Victoria (CCV) FFQ to collect data on participants’ habitual diet^(33)^. This FFQ is a well-established and validated tool for evaluating an individual’s typical diet with its validity demonstrated in the Australian population^(34)^. Participants were asked to report on the frequency of consumption and portion sizes of seventy-four food items and six types of alcoholic beverages over the past 12 months^(33)^. The FFQ was self-administered and took approximately 20–30 min to complete. The participants completed the questionnaire during their first study visit.

The raw data, including frequency and portion sizes, were used to calculate daily intake in grams. Nutrient and energy intake were calculated by multiplying the frequency of consumption of each item by its nutrient content. Nutrient values were calculated using nutrient composition data sourced from the AUSNUT 2007. Additionally, the data on total energy intake in KJ, calculated grams per day, and frequency of consumption of the seventy-four items and six beverages were extracted for every participant.

##### European Prospective Investigation into Cancer and Nutrition FFQ

The European Prospective Investigation into Cancer and Nutrition (EPIC) FFQ^(35)^ was utilised in the CANN trial to gather information about participants’ dietary habits. The EPIC FFQ is a well-established and validated tool for evaluating an individual’s typical diet, which is valid in various European populations^(35)^. Each participant completed a self-administered questionnaire at their baseline visit, which took 30–60 min to complete. The FFQ captures data on the typical frequency of consumption (through a nine-point scale) of 146 items over the previous 12 months, along with information gathered on dietary supplements and cooking and consumption practices^(35)^. The specified servings are indicated using units, commonly consumed portions, or household measures.

The raw data, including frequency and portion sizes, were used to calculate daily intake in grams. Energy and nutrient intakes were calculated by multiplying the frequency of consumption of each item by its nutrient content. Furthermore, data on total energy intake in KJ, calculated grams per day, and frequency of the 146 items were extracted for every participant.

#### Dietary patterns

In Table 5, a comprehensive overview of the MeDi, DASH and MIND diet scores is provided, which were scored according to the methods proposed by Martinez-Gonzales et al. (2012), Folsom et al (2017) and Morris et al. (2015), respectively. The table provides relevant information on the specific questions or food components per category, the amount of grams equivalent to a serving and the thresholds per food component, which are dichotomous for the MeDi and trichotomous for both the DASH and MIND.

**Table 5.** Gives an overview of the MeDi, DASH and MIND diet scores

	MeDi – Martinez-Gonzalez et al. (2012)				DASH – Folsom et al. (2007)				MIND – Morris et al. (2015)			
	Questions	Servings per day	Score	Grams equivalent of a serve	Component	Servings per day	Score	Grams equivalent of a serve	Components	Servings per day	Score	Grams equivalent of a serve
Olive oil and fats	Olive oil (%)	No	0	50 % of average intake per day from fats and oils should come from olive oil	% kcal from fat	≥ 33	0	Consumed fat or saturated fat in grams *37·7/total energy intake (kJ) × 100	Olive oil*	Not primary oil	0	> = 50 % of average intake
		Yes	1			> 30 to < 33	0·5			Primary oil	1	
						≤ 30	1					
	Olive oil	< 13·5 g	0	13·5 g	% kcal from SFA	≥ 13	0					
		≥ 13·5 g	1			> 10 to < 13	0·5					
						≤ 10	1					
Vegetables and fruits	Vegetables	< 2	0	200 g	Vegetable	< 2	0	Green leafy veg, stalk vegetables = 35 g; other veg = 70 g; vegetable juice, dips and soup = 120 g	Green leafy vegetables	≤ 0·29	0	Lettuce = 35 g
		≥ 2	1			≥ 2 tο < 4	0·5			> 0·29 to < 0·86	0·5	
						≥ 4	1			≥ 0·86	1	
	Fruit	< 3	0	150 g	Fruits	< 2	0	Banana = 118 g; apple = 182; medium fruits (grapefruit, orange, grapes, melon, peaches, pears, plum, passionfruit, kiwi, etc) = 150 g; dried fruit = 28 g; berries = 67 g; canned fruit = 113 g; fruit and juice = 124 g	Other vegetables	< 0·71	0	Other veg = 70 g; vegetable juice, dips and soups = 120 g
		≥ 3	1			≥ 2 tο < 4	0·5			≥ 0·71 tο < 1	0·5	
						≥ 4	1			≥ 1	1	
									Berries	< 0·14	0	Blackberry, cranberry, mulberry, raspberry, strawberry, goji berry = 67 g
										≥ 0·14 tο < 0·29	0·5	
										≥ 0·29	1	
Meat	Red meat, hamburger or meat products	> 1	0	100 g	Meats, poultry and fish	≥ 4	0	Cooked meats, poultry or fish = 85 g; 1 egg and savoury eff dishes = 50 g	Red meat and products	≥ 1	0	Cooked meat = 85 g
		≤ 1	1			> 2 to < 4	0·5			≥ 0·57 tο < 1	0·5	
						≤ 2	1			< 0·57	1	
	Chicken, turkey or rabbit meat (% of total meat intake)	≤ 50	0	> 50 % (Preference)					Poultry	< 0·14	0	Cooked poultry = 85 g
		> 50	1							≥ 0·14 tο < 0·29	0·5	
										≥ 0·29	1	
	Fish or shellfish	< 0·43	0	125 g of fish or 4–5 units or 200 g of shellfish					Fish	< 0·033	0	Cooked fish = 85 g
		≥ 0·43	1							≥ 0·033 tο < 0·14	0·5	
										≥ 0·14	1	
Dairy	Butter, margarine or cream	> 1	0	12 g	Dairy	< 1	0	Milk or yogurts = 246 g; cheese = 42·5 g*; creams and dairy product-based savoury sauses = 214 g*; dishes with milk as major component (ice cream, custard, pudding, cheesecake, trifle, tiramisu, iced coffee, milkshake, milk-based fruit drinks) = 246 g	Butter, margarine	> 2	0	Butters, margarine and table spreads = 14 g
		≤ 1	1			≥ 1 tο < 2	0·5			≥ 1 to ≤ 2	0·5	
						≥ 2	1			< 1	1	
									Cheese	≥ 1	0	Cheeses and cheese spreads = 42·5 g
										≥ 0·14 tο < 1	0·5	
										< 0·14	1	
Legumes and nuts	Legumes	< 0·43	0	150 g	Nuts, seeds and dry beans	< 0·29	0	Nuts and seeds = 42·5 g; peanut butter = 30 g; legumes, beans, peas and products (hummus, miso, chickpea/soya flour, backed beans) = 98 g*	Beans	< 0·14	0	Legumes, lentels and products (temphe, tofu, legume dips) = 98 g*
		≥ 0·43	1			≥ 0·29 tο < 0·57	0·5			≥ 0·14 tο ≤ 0·43	0·5	
						≥ 0·57	1			> 0·43	1	
	Nuts	< 0·43	0	30 g					Nuts	< 0·033	0	Nuts and seeds = 42·5 g
		≥ 0·43	1							≥ 0·033 tο < 0·71	0·5	
										≥ 0·71	1	
Grains					Total grains	< 5	0	Bread = 43 g; cooked rice, pasta, quinoa = 100 g; porridge = 110 g; breakfast cereal (all bran, brand flakes, weet bix, cornflakes) = 26 g; flour, uncooked grains (pasta, rice, oats, etc), cereal products, muesli bar, muelsi, cakes, savoury biscuits, cracker, popcorn, pancakes, scone, waffle = 57 g*	Whole grains	< 1	0	Bread = 43 g; uncooked pasta, dry cereal = 38 g; cooked cereal/porridge = 180 g; cooked rice, noodles, pasta, bulgar, quinoa = 133 g; other (flour, popcorn, muesli and cereal-style bars, savoury biscuits, crispbread) = 57 g*
						≥ 5 tο < 7	0·5			≥ 1 tο < 3	0·5	
						≥ 7	1			≥ 3	1	
					Whole grains	< 1	0					
						≥ 1 tο < 2	0·5					
						≥ 2	1					
Discretionary food and drinks	Sweet/carbonated beverages	≥ 1	0	Times/day	Sweets	≥ 1·14	0	Sugar and syrups, icing, spreads (jelly, jam) = 18 g; sorbet and gelatine dessert = 119; soft drinks = 372 g; other (ice cream, chocolate, flavoured milk drinks, sweet biscuits, cakes, desserts, buns, crumpets, wafels, etc.) = 100 g*	fast/fried foods	≥ 0·57	0	Times/day
		< 1	1			> 0·71 to < 1·14	0·5			≥ 0·14 tο < 0·57	0·5	
						≤ 0·71	1			< 0·14	1	
	Commercial sweets or pastries (cakes, cookies, biscuits and custard)	≥ 0·43	0	Times/day					Pastries, sweets	≥ 1	0	Sugar and syrups, icing, spreads (jelly, jam, marmelade) = 18 g; sorbet and gelatine dessert = 119; milkshake = 152 g; soft drink = 372 g; other (pavlova, cakes, sweet buiscuits, chocolate, muffins, scones, brownie, crap, waffle, etc) = 100 g*
		< 0·43	1							≥ 0·71 tο < 1	0·5	
										< 0·71	1	
Alcohol	Wine	< 1	0	150 ml					Wine	> 1 or 0	0	141·75 g
		≥ 1	1							> 0 to < 1	0·5	
										1	1	
Other	Vegetables, pasta, rice or other dishes seasoned with sofrito	< 0·29	0	Times/day	Na	> 2401	0	mg/day				
		≥ 0·29	1			> 1500 to ≤ 2401	0·5					
						≤ 1500	1					
Total Score			14				11				15	

NA, not applicable; DASH, Dietary Approaches to Stop Hypertension; MeDi, Mediterranean diet; MIND, Mediterranean-DASH Intervention for Neurodegenerative Delay.

Table organisation: the table includes information on the specific questions or food components per category, the amount of grams equivalent to a serving, the cut-off points per food component which are dichotomous (MeDi) or trichotomous (DASH and MIND). Lastly, the highest total score is presented in the last row.

* Average.

##### Mediterranean diet dietary pattern

The MeDi is a traditional dietary pattern that is commonly consumed by individuals living around the Mediterranean Sea. It places a strong emphasis on the use of olive oil as the primary source of fat, as well as incorporating vegetables, fruits and legumes into daily meals. Fish and wine are consumed in moderation, while red meat and processed foods are kept to a minimum^(36)^. Adherence to this dietary pattern was evaluated using the fourteen-item Mediterranean Diet Adherence Screener (MEDAS) by Martinez-Gonzalez et al. (2012), which assigns scores of either 1 or 0 to 14 dietary components^(37)^. The current paper utilised the Mediterranean Diet Adherence Screener scoring instrument as it is a validated and brief measure with broad applicability across diverse demographic groups^(37)^. Its avoidance of population-specific median cut-offs increases its suitability for standardisation in diverse settings^(8)^. Four questions inquire about food habits and frequency of consumption, while the remaining ten inquire about the amount of consumption per day or week. For example, some questions ask about topics such as the use of olive oil as the main culinary fat, preference for chicken, turkey or rabbit over red meat, frequency of carbonated or sugar-sweetened beverage consumption and weekly servings of legumes, fish, shellfish and nuts^(15)^. To obtain a final score ranging from 0 to 14, the fourteen items are summed, with higher scores indicating better adherence to the MeDi, as noted by Martínez-González and colleagues (2012). However, due to the lack of information on olive oil in both FFQ, and the absence of data on Sofrito consumption in the EPIC FFQ, the maximum score for the CCV FFQ is 12, while the maximum score for the EPIC FFQ is 11 which are large methodological limitations for the computation of the MeDi score. The determination of olive oil as the primary culinary fat source from the ASA24 involved calculating the ratio of the amount of olive oil consumed to the total fat content, expressed as a percentage. The score for the MeDi is presented in Table 5, which has been slightly modified from the original score by Martinez-Gonzalez et al. (2012). The components of the score have been converted to servings per day. While the MEDAS questionnaire is usually employed directly, in this case, data collected from other dietary assessment tools such as the ASA24, CCV FFQ and EPIC FFQ were used to score adherence to the MeDi. The specific included items from the ASA24, CCV and EPIC FFQ, integral to scoring MeDi adherence, are outlined in Table 2.

##### Dietary Approaches to Stop Hypertension dietary pattern

The DASH dietary pattern is a recommended diet for reducing cardiovascular risk factors such as hypertension and low-density lipoprotein cholesterol, which are associated with dementia (Appel et al., 1997; Most-Windhauser, 2001). Current guidelines that aim to prevent cardiovascular risk factors emphasise lifestyle modifications and advocate for adherence to the DASH diet, which emphasises high consumption of fruits, vegetables, grains, nuts and low-fat dairy products while reducing the consumption of sweets, SFA, sugar-containing beverages and Na to lower high blood pressure^(17)^. The constructed DASH index by Folsom et al. (2007) was used to assess adherence to the DASH low-NA diet^(17)^. The DASH dietary pattern score includes eleven components, with final scores ranging from 0 to 11 obtained by summing these items. Higher scores reflect better adherence to the DASH diet. Table 5 presents the DASH score, while the specific included items from the ASA24, CCV or EPIC FFQ are outlined in Table 3. Table 5 has been modified from the original DASH index score by Folsom et al. (2007) to present the components in servings per day when possible. Most of the components assessed the servings consumed per day, such as total grain intake, whole grain intake, vegetable intake, fruits, dairy foods, meats/poultry and fish and intake of nuts, seeds, dry beans and sweets. One component assessed Na consumption based on the number of milligrams consumed per day, while two components evaluated the percentage of energy content consumed from fats and saturated fatty acids. The percentage of fat and saturated fats consumed was calculated by multiplying the number of grams consumed, by 37·7 to convert the values from grams to kilojoules and dividing it by the total energy consumed. Serving sizes, which were not mentioned in the paper by Folsom et al. (2007), were extracted from the USA Department of Agriculture National Nutrient Database for Standard Reference dietary guidelines (2015–2020), as the original DASH dietary pattern was scored in USA serving sizes. The serving size for alcohol was extracted from the National Indigenous Australians Agency website^(38)^.

##### Mediterranean-DASH Intervention for Neurodegenerative Delay dietary pattern

The MIND diet has been created by investigators at Rush University and is styled after the MeDi and DASH diet. The MIND diet differentiates from the MeDi, and DASH diet in the number of servings of fish and dairy product and emphasises the intake of green leafy vegetables and berries. These modifications were made to align with evidence that showed neuroprotective effects^(39)^. For example, the servings of fish are much lower in the MIND diet compared with the MeDi and DASH diet, as there is evidence that one meal per week is sufficient to lower the risk of dementia.^(40–42)^. The MIND consists of fifteen components, with a key focus on promoting the intake of ten food components. These include green leafy vegetables, other vegetables, berries, fish, poultry, beans, nuts, olive oil and wine. Simultaneously, the diet emphasises a reduction in the consumption of five unhealthy food components, namely red meats, fast and fried food, butter and margarine, cheese, as well as pastries and sweets^(18)^. A combination of the constructed MIND scores by Morris et al. (2015) and Meuller et al. (2020) was used to assess adherence to the MIND^(43)^. Table 5 presents the MIND score, while the specific included items from the ASA24, CCV FFQ or EPIC FFQ are outlined in Table 4. To obtain a final score ranging from 0 to 15, the fifteen items are summed, with higher scores indicating better adherence to the MIND. However, due to the lack of information on olive oil in both FFQ, the maximum score for the CCV FFQ and EPIC FFQ got reduced to 14. The presented MIND score in Table 5 has been slightly modified from the original MIND score by Morris et al. (2015), as the components are presented in servings per day when possible. If the serving sizes of an item included in the MIND was not specified in the paper by Morris et al. (2015) or Meuller et al. (2020), they were extracted from the USA Department of Agriculture National Nutrient Database for Standard Reference dietary guidelines (2015–2020). These guidelines were used because the original MIND dietary patterns were scored in USA serving sizes. The serving size for alcohol was extracted from the National Indigenous Australians Agency website^(38)^. One serving of alcohol was calculated by dividing the amount in grams consumed by 141·748 g (5 ounces). To calculate the consumed frequency of fried food, the amount consumed each month was divided by 30·417 days. Last, olive oil used as primary oil has been calculated by dividing the amount of olive oil consumed by the total amount of fat times 100 %.

#### Scoring process

**Fig. 1.**
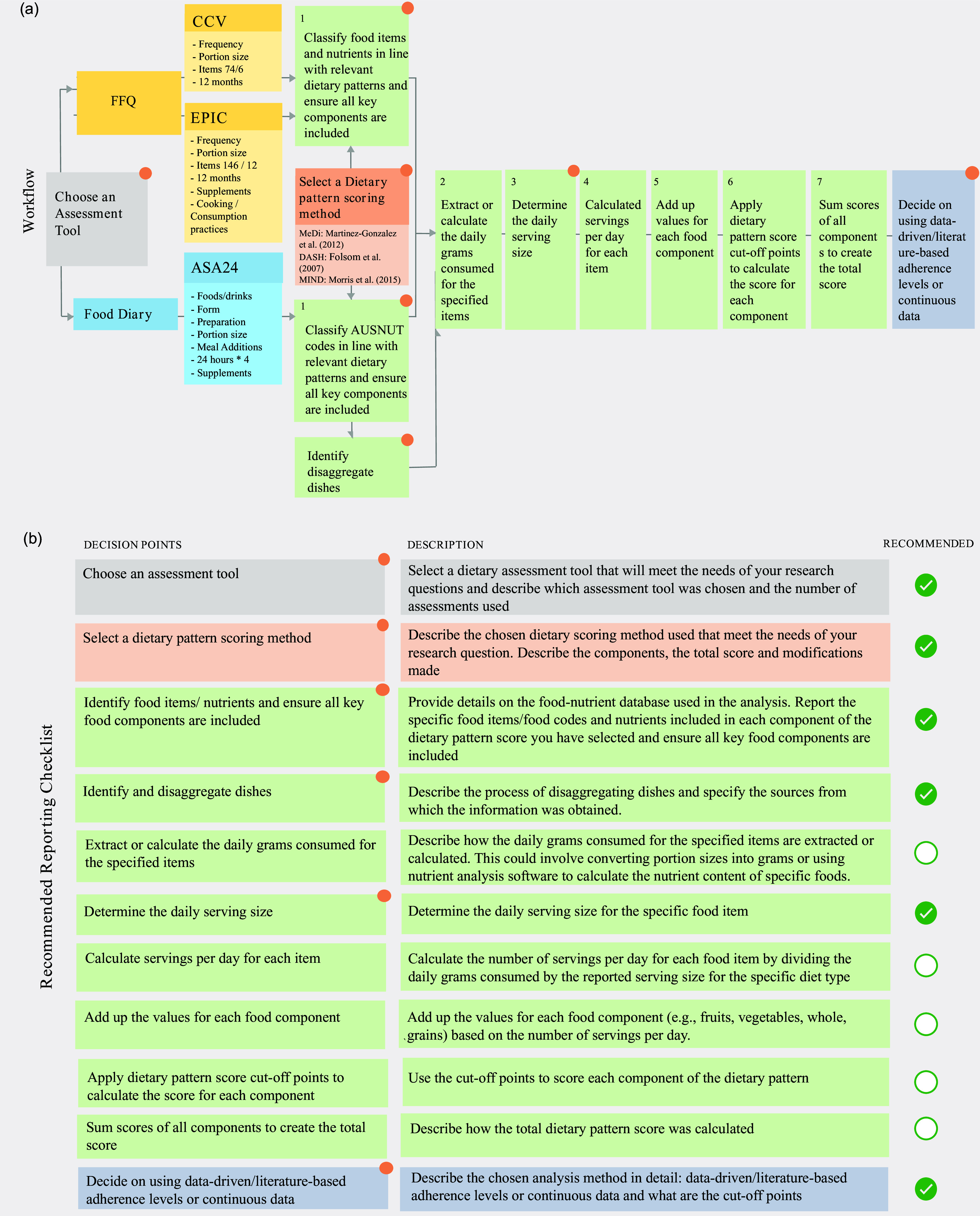
(a) Dietary pattern scoring workflow described in this paper, from assessment tool selection to choosing the number of cut-off points for the analysis and the use of absolute or data-driven tertiles. The workflow starts with choosing an assessment tool and the dietary pattern scoring method, after which (1) Relevant items from the ASA24, CCV FFQ and EPIC FFQ were chosen, (2) daily grams consumed for selected items were extracted, (3) daily serving size in grams was determined using the chosen dietary pattern scoring method (if applicable), (4) daily servings consumed were calculated, (5) items per component were weighted and summed, (6) cut-off points were applied to score components and (7) component scores were summed to obtain each participant’s total diet score. Finally, decide between data-driven/literature-based adherence levels or continuous data and describe the corresponding cut-off points for the analysis. Key subjective choices are marked with a symbol: Choosing the assessment tool, dietary pattern scoring method, identifying food items, determining serving sizes and disaggregating dishes when exact matches are absent and choosing data analysis methods and cut-off points. (b) Presents the recommended reporting checklist, detailing crucial elements which require a description in future research articles.

To compute the MeDi, DASH and MIND score using the various assessment tools, we followed seven steps outlined in Fig. 1: (1) relevant items from the ASA24, CCV FFQ and EPIC FFQ were selected, ensuring the availability and inclusion of all key food components (included items for each assessment tool are presented in Table 2–4), (2) the daily grams consumed for the selected items extracted, (3) the daily serving size was determined, guided by Martinez-Gonzalez et al. (2012), Folsom et al. (2007) and Morris et al. (2015) (presented in Table 2–4 and 5), (4) the consumed servings per day were calculated for each item, (5) all items per component were weighted and summed to calculate a single value per food component and (6) the recommended daily servings which were assigned to a component score provided by Martinez-Gonzalez et al. (2012), Folsom et al. (2007) and Morris et al. (2015) were used to assign a full (1) or no (0) point for each component in the MeDi or a full (1), a half (0·5) or no (0) points for each component in the DASH and MIND pattern. Lastly, (7) the scores for each food component were summed to compute the total MeDi, DASH and MIND score for each participant. The scoring process for the individual diet scores can be found in online Supplementary Material B.

#### Analysis approach

After calculating the total scores for dietary patterns, one can evaluate adherence through the analysis of continuous variables or by forming groups based on data-driven or literature-based methods. In our current methodology, the cut-off points for tertiles are determined through a data-driven approach, with each tertile encompassing roughly 33·3 % of the participants, alongside a literature-based approach which uses the cut-off point provided by the original articles^(15,17,18)^. The tertiles and quintiles derived from both data-driven and literature-based methods are detailed in Table 6.

**Table 6.** Dietary pattern cut-off points in each clinical trial – literature-based and data-driven approach

		MeDi tertiles (Martinez-Gonzalez et al. (2012))			DASH quintiles (Folsom et al. (2007))					MIND tertiles (Morris et al. (2015))		
					Literature-Based							
Clinical trial		T1	T2	T3	Q1	Q2	Q3	Q4	Q5	T1	T2	T3
MAST	Range	2·0–7·0	8·0–9·0	≥ 10	0·5–3·5	4·0–4·0	4·5–5·0	5·5–6·0	6·5–10·0	2·5–6·5	7·0–8·0	8·5 − 10
	*n*	126	11	2	40	19	34	21	25	59	39	40
PLICAR	Range	0–7·0	8·0–9·0	≥ 10	0·5–3·5	4·0–4·0	4·5–5·0	5·5–6·0	6·5–10·0	2·5–6·5	7·0–8·0	8·5 − 10
	*n*	161	1	0	96	14	29	12	4	28	68	49
CANN-Melbourne	Range	0–7·0	8·0–9·0	≥ 10	0·5–3·5	4·0–4·0	4·5–5·0	5·5–6·0	6·5–10·0	2·5–6·5	7·0–8·0	8·5 − 10
	*n*	96	2	0	10	10	34	22	22	17	34	47
CANN-UK	Range	0–7·0	8·0–9·0	≥ 10	0·5–3·5	4·0–4·0	4·5–5·0	5·5–6·0	6·5–10·0	2·5–6·5	7·0–8·0	8·5−10
	*n*	144	1	0	11	14	43	35	41	21	51	72

					Data-Driven							
Clinical trial		T1	T2	T3	T1	T2	T3			T1	T2	T3
MAST	Range	2·0–5·0	6·0–6·0	7·0–10	1·5–4·0	4·5–5·5	6·0–8·5			3·0–6·0	6·5–8·0	8·5 − 13
	*n*	81	22	36	59	45	35			48	50	41
PLICAR	Range	0·0–3·0	4·0–4·0	5·0–8	0·0–3·0	3·5–4·0	4·5–7·5			4·0–7·0	7·5–8·0	8·5–11
	*n*	81	41	34	71	40	45			60	47	49
CANN-Melbourne	Range	0·0–3·0	4·0–4·0	5·0–9	2·5–4·5	5·0–5·5	6·0–9·0			4·0–7·5	8·0–9·0	9·5–11·5
	*n*	50	30	18	37	32	29			36	41	21
CANN-UK	Range	0·0–3·0	4·0–4·0	5·0–8	2·0–5·0	5·5–6·0	6·5–9·5			2·5–6·5	7·0–8·0	8·5–11·5
	*n*	84	31	29	68	35	41			51	55	38

*n,* number of participants; Q, quintile; T, tertiles.

Ranges and participant distribution are provided for each adherence group within the respective clinical trials. Literature-based cut-offs are based on the dietary patterns as defined in the specified literature sources (MeDi by Martinez-Gonzalez et al. (2012), DASH by Folsom et al. (2007), MIND by Morris et al. (2015)). Data-driven cut-off values for tertiles are determined through data-driven analysis.

## Results

The method section outlines the dietary pattern scoring workflow, visually represented in Fig. 1(a). This process encompasses various steps, starting with the selection of an assessment tool and concluding with the determination of data-driven or literature-based cut-off points. Throughout this analysis process, it became evident that several steps need subjective choices from the researcher, highlighting the need for detailed reporting among future research articles. To promote comprehensive and accurate reporting among future researchers, a recommended reporting checklist (Fig. 1(b)) was crafted based on the outlined workflow in the method section. This checklist can also be valuable for reviewers evaluating research articles.

The recommended reporting checklist specifically underscores points in the workflow where subjective choices by the researcher introduce individual differences between studies as illustrated in Fig. 1(b). This involves specifying details such as the assessment tool used, the included number of items and the number of assessments it includes. Additionally, details on the scoring method are required, including the total score, and any modifications. Due to variability among assessment tools, the checklist also demands a description of included food and beverage items in each dietary pattern component. Further, verification of the inclusion of all key food components is emphasised. Daily serving sizes for each item need to be provided, as the serving size used for calculating servings per day for each item is absent in the original papers. Lastly, the checklist underscores the importance of describing the adherence levels, whether continuous, data-driven or literature-based. When employing data-driven cut-off points, explicit clarification of distinctions from literature-based cut-off points is considered insightful. Such comprehensive reporting enhances researchers’ understanding of result interpretation and facilitates evidence synthesis into dietary guidelines or interventions. This checklist can be supplemented by the Strengthening the Reporting of Observational Studies in Epidemiology-nutritional epidemiology guidelines^(23)^.

Table 6 provides an overview of the number of participants included in adherence groups using both the literature-based and data-driven approaches. The data-driven and literature-based adherence levels for the MeDi, as outlined by Martinez-Gonzalez et al. (2012), DASH presented by Folsom et al. (2007) and for the MIND presented by Morris et al. (2015) are presented in Table 6. The literature-based approach resulted in unevenly weighted groups when assessing adherence to MeDi and DASH. Particularly, the MeDi groups were notably skewed, with almost all participants adhering to the lowest tertile group. In contrast, the data-driven approach, while yielding different cut-off points for each clinical trial, provided a more balanced distribution.

Supporting analyses were conducted to determine whether adherence to the MeDi, DASH and MIND dietary pattern scores through data-driven and literature-based approaches were associated with expected changes in biochemical markers and cardiovascular measures (Table S3 to S10 of online Supplementary Material C).

## Discussion

The purpose of this study was to further standardise the dietary pattern scoring process from the dietary assessment tools in order to enhance the reproducibility and comparability of their outcomes. The study achieved two important goals. First, a step-by-step reproducible workflow was documented that scores the MeDi, DASH and MIND pattern using three commonly used dietary assessment tools: 24-h recall and two variations of an FFQ. This detailed workflow will allow future studies to replicate these scoring procedures, promoting consistency and facilitating generalisability across studies, thereby allowing for direct comparison in future research. Second, since most studies do not fully report how dietary scores were created, it is difficult to compare results across studies. Therefore, this workflow helped produce a recommended reporting checklist based on points within the scoring procedure, which can influence outcomes, which when followed, will enhance the synthesis of evidence into dietary guidelines. The following section will discuss the identified limitations of the assessment tools used to score the MeDi, DASH and MIND dietary patterns. This will include an exploration of factors hindering reproducibility, an overview of the strategies implemented to mitigate these limitations and an assessment of potential future directions.

### Limiting factors of the assessment tools

Selecting an appropriate dietary assessment tool is a crucial choice that influences the amount of detailed information that can be collected in any research experiment and is therefore an important source of variability. For instance, FFQ typically have a predetermined list of food and beverage items, which might not be comprehensive enough to capture all the foods and beverages consumed by an individual. The CCV FFQ, for instance, collects data from seventy-four food items and six alcoholic beverages, and the EPIC FFQ collects data from 146 food items and twelve beverages, while the ASA24 allows for more flexibility in reporting as it can contain information on a total of 5740 foods and beverages.

The level of detail captured by the assessment tools used in this study had a significant impact on the highest achievable score of the dietary patterns as presented in Tables 2 and 4. The CCV and EPIC FFQ employed in this study are limited by their inability to capture certain dietary information, such as olive oil consumption. This lack of information affects the accuracy of adherence to the MeDi and MIND diet, reducing their highest achievable score from 14 to 12 and from 15 to 14, respectively. Additionally, the EPIC FFQ fails to capture information about how many times per week a participant consumes dishes seasoned with sofrito, further reducing the total score of the MeDi dietary pattern to 11. To address this major limitation, it is crucial to employ assessment tools that encompass all key components included in the dietary patterns, such as the ASA24, or utilize FFQ like the Harvard-Willett FFQ^(44)^, which provide a more accurate granular reflection of individuals’ actual consumption habits.

Another limitation that arose during the scoring process is that the MIND score utilised in this study is limited by its method of assigning a score of 1 to an exact serving of alcohol equivalent to 5 ounces, which fails to account for individuals who consumed slightly more or less than one glass of wine. This may lead to inaccurate assessments of alcohol consumption among study participants.

Further, it is important to note that assessing self-reported diet recall in participants with memory issues presents a limitation. As a result, FFQ may be subject to recall bias, leading to inaccuracies in the reported dietary information^(45)^. Further, FFQ are unable to capture day-to-day variations as they collect information on typical dietary intake over a 12-month period. In contrast, more detailed assessment tools such as the ASA24 collect information about all foods and beverages consumed during the previous day, including both weekdays and weekends, which provides a more accurate estimate of an individual’s day-to-day variations in dietary intake^(46)^.

To obtain enough detailed information on the different components of the MeDi, DASH and MIND dietary patterns, researchers are encouraged to carefully select a dietary assessment tool that aligns with the research question and can be standardised to obtain consistent and accurate results. Assessment tools such as the ASA24, dietary records and a 7-d food diary may overcome some of the limitations of the included FFQ, such as the limited food and beverage list, and the day-to-day variation^(47)^. However, it is important to note that these alternative tools may have their own limitations^(4,6,48)^. Nonetheless, the provided scoring workflow for the ASA24 can, however, be used to standardise the scoring process beyond the specific assessment tools included in the current paper.

### Limiting factors in the scoring process

An aspect where subjective judgement arises during the scoring process is during the identification of food items from the FFQ and ASA24 that fit into the different food component groups. Since this identification is subjective, it can result in differences in the amount and type of food items included. Another point where subjectivity can arise is during the identification and calculation of items from disaggregated dishes as not every dish is included in the AUSNUT recipe file, in some cases a similar dish needs to be matched. Additionally, identifying the serving sizes of all the individual items is also a subjective step, as the information on the grams equivalent to a serving is not always reported for each item in the utilised standardised dietary pattern methods. This lack of information on the number of grams equivalent to a serving size can cause variability and may present a challenge for accurate comparisons across studies. Further, the differences in servings stipulated in the FFQ and the differences in serving sizes between countries and regions increase this challenge^(49–51)^.

### Limiting factors in the analysis approach

Besides standardising the scoring process, it is crucial to consider standardising the analysis methods, as the choice between continuous data and deciding whether to use data-driven or literature-based adherence level cut-off points introduces further variability. While literature-based cut-off points promote comparability and generalisability of findings across different populations and studies, they may not always be feasible. This was evident in our analysis, where the population adherence levels to the MeDi were consistently low across all our populations, deviating from the original paper^(15)^, which defines high adherence as a score of ≥ 10 out of 14. Notably, the total score was adjusted to 12 for the CCV FFQ due to its exclusion of information about olive oil and further reduced to 11 for the EPIC FFQ. As a result, none of the participants in the CANN and PLICAR trials attained a score ≥ 10. Even in the MAST population, only two participants achieved a MeDi score ≥ 10. This is despite the trial’s intentional recruitment strategy, aiming for diversity in diet quality by including 50 % of the population adhering to an ‘optimal’ diet and the other 50 % to a sub-optimal diet scored through the Diet Screening Tool^(25)^. While there were still only two participants in this trial with a MeDi score ≥ 10, geographical variations might also contribute to the observed low adherence, particularly given the documented low adherence to vegetables in Australian populations^(52)^. Furthermore, the distribution of participants across literature-based adherence groups for the DASH and MIND was more favourable and even better in the MAST trial compared with the CANN and PLICAR trials. When assessing data-driven cut-off points, which are useful in describing results within a specific population but may not be comparable across populations and studies^(3)^, it is essential to emphasise the differences between the obtained data-driven cut-off points and those described in the original papers. These differences in the analysis methods might contribute to the variations in effect estimates of a given intervention.

### Steps towards overcoming limitations in dietary pattern scoring

The current paper addresses some of these limitations by presenting a list of food items for each component of the dietary patterns available through the ASA24, the CCV FFQ and EPIC FFQ (outlined in Tables 2–4) and providing the utilised grams equivalent to a serving size for each item (outlined in Tables 4 and 5). Missing serving sizes from the utilised dietary pattern scoring methods^(17,18)^ were extracted from the USA Department of Agriculture National Nutrient Database for Standard Reference Dietary Guidelines (2015–2020) for the DASH and MIND dietary patterns. These databases were used because the original DASH and MIND dietary patterns were scored in USA serving sizes. However, certain items like beans, cheese and yoghurt, which are extensively detailed in the USA Department of Agriculture database (each of the fifteen types of beans had a distinct serving size with varying serving sizes), posed a challenge. In such cases, an average of all the diverse types of these items was utilised since the ASA24 and FFQ did not encompass all the diverse types of beans, cheeses and yoghurts. In addition, this paper includes a description of how food items were extracted from reported disaggregated dishes.

### Future directions

The scope of the current paper did not include evaluating the effects of various dietary pattern scoring methods to score the MeDi, DASH and MIND, despite there being several dietary pattern scoring methods for each. The MeDi, for instance, has twenty-eight unique scoring methods available^(9)^. However, given the current lack of consensus regarding the most appropriate scoring method, it is imperative that future research explores the impact of utilising different dietary pattern scoring methods. Identification of the most suitable dietary scoring method would stimulate future research to use a single of a small number of approaches and further standardise this field.

### Conclusion

This paper addresses the pressing issue of inconsistency in the dietary pattern scoring process and its reporting within the literature, which hinders the synthesis of consistent evidence into dietary guidelines. To address this challenge, a clear step-by-step reproducible workflow, along with a recommended reporting checklist, has been presented. However, it is essential to acknowledge that subjective judgements, such as the choice of analysis method, remain integral to the scoring process, underscoring the ongoing need for transparent reporting of employed methods. Lastly, the identified limitations in the assessment tools utilised in this paper offer valuable insights for future researchers, on what aspects to consider, when selecting assessment tools for scoring the MeDi DASH or MIND dietary patterns.

## Supporting information

Arnoldy et al. supplementary material Arnoldy et al. supplementary material

## Supplementary material

For supplementary material/s referred to in this article, please visit https://doi.org/10.1017/S0007114524000072
