# Towards consistency in dietary pattern scoring: standardising scoring workflows for healthy dietary patterns using 24-h recall and two variations of a food frequency questionnaire – CORRIGENDUM

**DOI:** 10.1017/S0007114524000680

**Published:** 2024-05-14

**Authors:** Lizanne Arnoldy, Sarah Gauci, Annie-Claude M. Lassemillante, Joris C. Verster, Helen Macpherson, Anne-Marie Minihane, Andrew Scholey, Andrew Pipingas, David J. White

The authors regret an error in the final title. The correct title is ‘Towards consistency in dietary pattern scoring: standardising scoring workflows for healthy dietary patterns using 24-h recall and two variations of a food frequency questionnaire’.

The article has been corrected.
