# Supplementary material for: Towards consistency in dietary pattern scoring: standardising scoring workflows for healthy dietary patterns using 24-h recall and two variations of a food frequency questionnair
Source: Br J Nutr. 2024 Jan 16;131(9):1554–77. doi: 10.1017/S0007114524000072 (PMC11043911; doi:10.1017/S0007114524000072)
Supplement: Arnoldy et al. supplementary material [file S0007114524000072sup001.docx]

# Context

[Supplementary Material A: 2](#_Toc152246299)

[Supplementary Material B: 9](#_Toc152246300)

[Scoring method MeDi 9](#_Toc152246301)

[Scoring method DASH 9](#_Toc152246302)

[Scoring method MIND 10](#_Toc152246303)

[Supplementary Material C: 11](#_Toc152246304)

[Measures 11](#_Toc152246305)

[Biochemical Markers and Cardiovascular measures 11](#_Toc152246306)

[Biochemical Markers 11](#_Toc152246307)

[Cardiovascular measures 11](#_Toc152246308)

[Supporting Analysis 12](#_Toc152246309)

[Covariates 13](#_Toc152246310)

[Supporting Results 13](#_Toc152246311)

[Sample Characteristics 13](#_Toc152246312)

[Biochemical Markers and Cardiovascular Outcomes 17](#_Toc152246313)

# Supplementary Material A:

| Table S1: Recorded eight-digit codes and the associated dishes from the ASA24 in the MAST dataset | |
| --- | --- |
| 8 Item |  |
| 12304001 | Bread or bread roll, topped/mixed with cheese |
| 12304002 | Bread or bread roll, topped/mixed with cheese & bacon |
| 12304007 | Bread or bread roll, topped/mixed with olives |
| 12404001 | Pasta, filled with meat, fresh, commercial, boiled, without added sauce |
| 12404004 | Pasta, filled with vegetables, fresh, commercial, boiled, without added sauce |
| 12509001 | Breakfast cereal, wheat bran, flakes, sultanas, added vitamins B1, B2, B3, B6 & folate, Fe & Zn |
| 12509007 | Breakfast cereal, whole wheat, flakes, dried fruit & nuts, added fibre, vitamins B1, B2, B3 & folate, Ca & Fe |
| 13303016 | Cake or cupcake, berry, undefined fat, uniced |
| 13304008 | Muffin, cake-style, berry, homemade from basic ingredients, uniced |
| 13304018 | Muffin, cake-style, raspberry & white chocolate, commercial, uniced |
| 13306004 | Slice, brownie, chocolate, with nuts, homemade from basic ingredients, fat not further defined |
| 13404004 | Quiche, Lorraine, homemade |
| 13404006 | Quiche, mixed vegetables, homemade |
| 13404009 | Quiche, salmon, or tuna, commercial |
| 13405001 | Dim sum, vegetable & meat filling, purchased frozen, microwaved, or steamed |
| 13405002 | Dumpling or wonton, savoury, chicken, steamed, boile,d or microwaved |
| 13405003 | Dumpling or wonton, savoury, meat, steamed, boiled, or microwaved |
| 13405004 | Dumpling or wonton, savoury, meat & seafood, steamed, boiled, or microwaved |
| 13405005 | Dumpling or wonton, savoury, seafood, steamed, boiled, or microwaved |
| 13405006 | Dumpling or wonton, savoury, vegetable, steamed, boiled, or microwaved |
| 13405010 | Pastry, filled with spinach & cheese, from frozen, baked, no added fat |
| 13405013 | Pasty, filled with vegetables, homemade from basic ingredients |
| 13405016 | Pie, savoury, chicken & vegetable, commercial |
| 13405018 | Pie, savoury, meat, commercial |
| 13405020 | Pie, savoury, meat, from frozen, baked or microwaved |
| 13405026 | Pie, savoury, meat filling, topped with potato, commercial |
| 13405030 | Pie, savoury, spinach & cheese, commercial |
| 13405033 | Pie, savoury, without pastry, meat filling, topped with mashed potato & cheese, homemade from basic ingredients |
| 13405035 | Sausage roll, commercial, ready to eat |
| 13405036 | Sausage roll, from frozen, baked or microwaved |
| 13405039 | Spring roll or rice paper roll, chicken & vegetable filling, fresh |
| 13405042 | Spring roll or rice paper roll, vegetable filling, fresh |
| 13406002 | Curry puff, beef & vegetable, deep fried, commercial |
| 13406004 | Dim sum, vegetable &/or meat filling, takeaway style, deep fried |
| 13406006 | Dumpling or wonton, savoury, meat, fried |
| 13406008 | Dumpling or wonton, savoury, seafood, fried |
| 13406011 | Samosa, vegetable, deep fried |
| 13406012 | Spring roll, meat &/or vegetable, purchased frozen, baked |
| 13406015 | Spring roll, meat & vegetable filling, fried, homemade from basic ingredients |
| 13406016 | Spring roll, vegetable filling, takeaway style, deep fried |
| 13501005 | Pizza, chicken & bacon, thick base, BBQ sauce, takeaway style or homemade |
| 13501013 | Pizza, ham & pineapple, purchased frozen, baked |
| 13501014 | Pizza, ham & pineapple, thick base, takeaway style & homemade |
| 13501015 | Pizza, ham & pineapple, thin base, takeaway style |
| 13501022 | Pizza, pepperoni, thick base, takeaway style & homemade |
| 13501023 | Pizza, pepperoni, thick base, fast food chain |
| 13501025 | Pizza, prosciutto, thick base, takeaway style & homemade |
| 13501031 | Pizza, supreme, thick base, takeaway style & homemade |
| 13501033 | Pizza, supreme, thin base, fast food chain |
| 13501036 | Pizza, vegetable, thick base, takeaway style & homemade |
| 13501037 | Pizza, vegetable, thin base, takeaway style |
| 13501040 | Pizza, vegetable, flat bread base, homemade |
| 13502002 | Pizza, supreme, thin base, takeaway style |
| 13502005 | Pizza, cheese & tomato, thin base, takeaway style |
| 13502009 | Pizza, pepperoni, purchased frozen, baked |
| 13502014 | Pizza, chicken & vegetable, thin base, takeaway style |
| 13502019 | Pizza, supreme, flat bread base, homemade |
| 13503001 | Filled bread roll, Asian-style, pork, mayonnaise, pate & vegetables |
| 13503021 | Filled bread roll, commercial, ham & salami, cheese, water-based sauce |
| 13503039 | Hot dog, bread roll, Frankfurt, onion & sauce filling |
| 13503043 | Kebab wrap, beef, with salad & sauce, takeaway |
| 13503046 | Kebab wrap, chicken, with salad & sauce, takeaway |
| 13503048 | Kebab wrap, falafel, with salad & sauce, takeaway |
| 13503049 | Kebab wrap, lamb, with salad, takeaway |
| 13503050 | Kebab wrap, lamb, with salad & sauce, takeaway |
| 13503063 | Sandwich or roll, filled with salad |
| 13503067 | Sandwich or roll, not further defined |
| 13505015 | Chicken burger, white roll, crumbed chicken breast, with lettuce & mayonnaise, fast food chain |
| 13505021 | Chicken roll, white roll, chicken, with mayonnaise, fast food chain |
| 13505022 | Fish burger, with cheese, fast food chain |
| 13505027 | Hamburger, white roll, beef patty, with cheese, lettuce, sauce, fast food chain |
| 13505029 | Hamburger, white roll, beef patty, with pickles, tomato sauce & mustard, fast food chain |
| 13505030 | Hamburger, white roll, 2 beef patties, with lettuce, onion, pickles, tomato, mayonnaise & sauce, fast food chain |
| 13505049 | Muffin, English style, with beef sausage patty, cheese & egg, fast food chain |
| 13506002 | Hamburger, white roll, beef patty, with cheese, onion, pickles & sauce, fast food chain |
| 13506004 | Hamburger, white roll, 2 beef patties, with bacon, cheese, lettuce, tomato & mayonnaise, fast food style |
| 13507002 | Chicken wrap, white tortilla, coated chicken breast strips, with salad & mayonnaise, fast food chain |
| 13507016 | Mexican wrap, bean, with cheese, salad & salsa |
| 13507018 | Mexican wrap, beef & bean, with cheese, salad & salsa |
| 13507019 | Mexican wrap, beef, with cheese & salsa |
| 13507020 | Mexican wrap, beef, with cheese, salad & salsa |
| 13507025 | Mexican wrap, beef & bean, with cheese, guacamole, salad & salsa |
| 13507027 | Mexican wrap, chicken, with cheese & salsa |
| 13507028 | Mexican wrap, chicken, with cheese, salad & salsa |
| 13507035 | Mexican wrap, fish, with cheese, guacamole, salad & salsa |
| 13507036 | Mexican wrap, with cheese, guacamole, salad & salsa |
| 13508004 | Mexican nachos, corn chips, beef, with cheese, guacamole & salsa |
| 13508008 | Mexican nachos, corn chips, with cheese & salsa |
| 13509004 | Frozen meal, beef lasagne, energy-controlled portion |
| 13509007 | Lasagne (Lasagne), beef, no added vegetables, homemade |
| 13509008 | Lasagne (Lasagne), beef, with added vegetables, homemade |
| 13509011 | Macaroni & cheese, homemade, cooked unfilled pasta, homemade cheese sauce |
| 13509013 | Pasta dish, commercial, cooked unfilled pasta, beef Bolognese sauce |
| 13509015 | Pasta dish, homemade, cooked unfilled pasta, commercial beef Bolognese sauce |
| 13509016 | Pasta dish, homemade, cooked unfilled pasta, commercial beef Bolognese sauce & added vegetables |
| 13509017 | Pasta dish, homemade, cooked unfilled pasta, homemade beef Bolognese sauce |
| 13509018 | Pasta dish, homemade, cooked unfilled pasta, homemade beef Bolognese sauce & added vegetables |
| 13509027 | Pasta dish, homemade, cooked unfilled pasta, commercial dairy-based sauce, added vegetables |
| 13509031 | Pasta dish, homemade, cooked unfilled pasta, homemade dairy-based sauce, added seafood, with or without added vegetables |
| 13509035 | Pasta dish, cooked unfilled pasta, oil based sauce, added chicken, with or without added vegetables |
| 13509042 | Pasta dish, commercial, cooked unfilled pasta, tomato-based sauce, added vegetables |
| 13509043 | Pasta dish, homemade, cooked unfilled pasta, commercial tomato-based sauce |
| 13509045 | Pasta dish, homemade, cooked unfilled pasta, commercial tomato-based sauce, added chicken, with or without added vegetables |
| 13509047 | Pasta dish, homemade, cooked unfilled pasta, commercial tomato-based sauce, added vegetables |
| 13509048 | Pasta dish, homemade, cooked unfilled pasta, homemade tomato-based sauce |
| 13509049 | Pasta dish, homemade, cooked unfilled pasta, homemade tomato-based sauce, added bacon, with or without added vegetables |
| 13509050 | Pasta dish, homemade, cooked unfilled pasta, homemade tomato-based sauce, added chicken, with or without added vegetables |
| 13509053 | Pasta dish, homemade, cooked unfilled pasta, homemade tomato-based sauce, added salmon or tuna, with or without added vegetables |
| 13509054 | Pasta dish, homemade, cooked unfilled pasta, homemade tomato-based sauce, added vegetables |
| 13509056 | Pasta dish, cooked unfilled pasta, homemade tomato-based sauce, added meat |
| 13509057 | Pasta dish, cooked filled pasta, beef Bolognese sauce |
| 13509062 | Pasta dish, cooked filled pasta, tomato-based sauce |
| 13509071 | Pasta in tomato-based sauce, prepared from dry mix with water & margarine spread |
| 13509072 | Spaghetti in meat sauce, canned, regular |
| 13509073 | Spaghetti in tomato & cheese sauce, canned, regular |
| 13509075 | Stir-fry, commercial, noodles, added chicken & vegetable |
| 13509077 | Stir-fry, commercial, rice noodles, added prawn, egg & vegetable (pad Thai) |
| 13509078 | Stir-fry, commercial, rice noodles, added prawn, pork, egg & vegetable (mee grub) |
| 13510002 | Pasta dish, homemade, cooked unfilled pasta, homemade carbonara sauce, added bacon, with or without added vegetables |
| 13511002 | Paella, white rice, with chicken, chorizo, mixed vegetables & seafood |
| 13511004 | Rice, brown, fried, no meat or vegetables, oil not further defined, homemade |
| 13511006 | Rice, brown, fried with chicken, egg & mixed vegetables |
| 13511008 | Rice, white, fried, no meat or vegetables, oil not further defined, homemade or commercial |
| 13511009 | Rice, white, fried with bacon or ham, egg & mixed vegetables |
| 13511011 | Rice, white, fried with bacon or ham, egg, prawns & vegetables |
| 13511012 | Rice, white, fried with beef, lamb, or pork & mixed vegetables |
| 13511013 | Rice, white, fried with chicken, egg & mixed vegetables |
| 13511016 | Rice, white, fried with egg & mixed vegetables |
| 13511019 | Rice, white, fried with mixed vegetables |
| 13511021 | Rice, white, pilaf style, with butter, stock & spices |
| 13511022 | Risotto, bacon, or ham & mixed vegetables, with cheese |
| 13511023 | Risotto, beef, lamb, or pork & mixed vegetable, with cheese |
| 13511024 | Risotto, chicken & mixed vegetables, with cheese |
| 13511025 | Risotto, mixed vegetables, with cheese |
| 13511026 | Risotto, mushroom, with cheese |
| 13511027 | Risotto, plain, with cheese |
| 13513001 | Gnocchi, potato, commercially prepared, boiled |
| 13513002 | Gnocchi, commercial or homemade, potato gnocchi, tomato-based sauce |
| 13513007 | Steamed bun, savoury, pork |
| 13514003 | Sushi, California roll, commercial |
| 13514004 | Sushi, chicken, with seaweed |
| 13514005 | Sushi, chicken & avocado, with seaweed |
| 13514006 | Sushi, chicken & avocado, without seaweed |
| 13514014 | Sushi, prawn tempura, with seaweed |
| 13514015 | Sushi, salmon & avocado, with seaweed |
| 13514016 | Sushi, salmon, with seaweed |
| 13514017 | Sushi, salmon, without seaweed |
| 13514018 | Sushi, tuna, with seaweed |
| 13514020 | Sushi, tuna & avocado, with seaweed |
| 13514022 | Sushi, vegetable, with seaweed |
| 13514023 | Sushi, not further defined |
| 13515003 | Couscous, homemade, cooked, with roast vegetables |
| 13515006 | Salad, couscous with cheese & vegetables |
| 15601004 | Casserole, homemade, salmon or tuna & vegetable, homemade dairy-based sauce |
| 15602007 | Tuna mornay, homemade, cooked unfilled pasta, homemade white sauce, cheese & breadcrumbs |
| 15603008 | Stir-fry, commercial, prawn, garlic sauce |
| 15603010 | Stir-fry, homemade, prawn, garlic sauce |
| 18701009 | Casserole, homemade, beef & vegetable, homemade tomato-based sauce |
| 18701010 | Casserole, homemade, beef, vegetable & legume, homemade sauce |
| 18701016 | Curry, homemade, beef, homemade sauce |
| 18701018 | Curry, homemade, beef & vegetable, homemade coconut milk-based sauce |
| 18701019 | Curry, homemade, beef & vegetable, homemade tomato-based sauce |
| 18701026 | Sauce, pasta, Bolognese, homemade using beef mince & homemade tomato-based sauce with vegetables |
| 18701029 | Stir-fry, commercial, beef & vegetable |
| 18701032 | Stir-fry, homemade, beef & vegetable |
| 18701034 | Stir-fry, homemade, beef & vegetable, commercial simmer sauce |
| 18702010 | Curry or casserole, homemade, beef & rice, not further defined |
| 18702012 | Frozen meal, beef & noodles or pasta, energy-controlled portion |
| 18702017 | Stir-fry, homemade, beef & vegetable, with noodles |
| 18703003 | Beef, schnitzel, breadcrumb coating, baked, roasted, fried, grilled or BBQed, olive oil |
| 18703012 | Meatball or rissole, beef, commercial, cooked |
| 18703020 | Meatball or rissole, beef mince, homemade, baked, roasted, fried, grilled or BBQed, fat not further defined |
| 18703021 | Meatloaf, homemade, beef |
| 18703022 | Rissole or patty, beef mince, grilled or fried, no added fat, fast food style |
| 18705002 | Casserole, homemade, lamb & eggplant, baked with homemade creamy sauce (moussaka) |
| 18705005 | Casserole, homemade, lamb & vegetable, homemade tomato-based sauce |
| 18705008 | Curry, homemade, lamb, homemade sauce |
| 18705010 | Curry, homemade, lamb & vegetable, homemade coconut milk sauce |
| 18705012 | Curry, homemade, lamb & vegetable, homemade tomato-based sauce |
| 18705014 | Frozen meal, lamb & vegetables, energy controlled portion |
| 18706002 | Curry, homemade, lamb & rice |
| 18707003 | Lamb, frenched cutlet/rack, fully-trimmed, breadcrumb coating, baked, roasted, fried, grilled or BBQ'd, fat not further defined |
| 18708007 | Stir-fry, commercial, pork, sweet & sour sauce |
| 18708011 | Stir-fry, homemade, pork & vegetable |
| 18709004 | Stir-fry, homemade, pork & vegetable, with rice or noodles |
| 18710002 | Pork, coated, baked, roasted, fried, grilled or BBQ'd, fat not further defined |
| 18712003 | Mixed dish, not further defined |
| 18901006 | Casserole, homemade, chicken & vegetable, homemade tomato based sauce |
| 18901008 | Curry, commercial, chicken, dairy based sauce, Indian |
| 18901009 | Curry, homemade, chicken, commercial sauce |
| 18901010 | Curry, homemade, chicken, homemade sauce |
| 18901012 | Curry, commercial, chicken & vegetable, coconut milk sauce |
| 18901014 | Curry, homemade, chicken & vegetable, homemade coconut milk sauce |
| 18901015 | Curry, homemade, chicken & vegetable, homemade dairy based sauce |
| 18901017 | Curry, homemade, chicken, vegetable & legume, homemade sauce |
| 18901021 | Stir-fry, commercial, chicken, satay sauce |
| 18901022 | Stir-fry, homemade, chicken |
| 18901023 | Stir-fry, homemade, chicken, commercial or homemade satay sauce |
| 18901024 | Stir-fry, commercial, chicken & vegetable |
| 18902006 | Crepe or pancake, savoury, filled with duck & vegetables, with sauce (Peking duck) |
| 18902009 | Curry, homemade, chicken & rice |
| 18902017 | Frozen meal, curry, chicken & rice, Indian style |
| 18902018 | Frozen meal, curry, chicken & rice, energy controlled portion |
| 18902019 | Frozen meal, vegetables & pasta or noodles, energy controlled portion |
| 18902024 | Stir-fry, homemade, chicken & vegetable, with noodles |
| 19204003 | Yoghurt, natural or Greek, high fat (~6%), added berry pieces |
| 19205003 | Yoghurt, berry pieces or flavoured, regular fat (~3%) |
| 19207002 | Yoghurt, berry pieces or flavoured, reduced fat (1%) |
| 19208002 | Yoghurt, berry pieces or flavoured, low fat (<0.5%) |
| 20701001 | Curry, commercial, tofu & vegetable |
| 20701003 | Stir-fry, homemade, tofu, commercial or homemade satay sauce |
| 20701004 | Stir-fry, homemade, tofu & vegetable |
| 21101003 | Soup, beef, vegetable & legume, prepared with stock or water, homemade from basic ingredients |
| 21101005 | Soup, broth with meat & noodles |
| 21101008 | Soup, chicken & vegetable, with grains or pasta, prepared with stock or water, homemade from basic ingredients |
| 21101009 | Soup, chicken, vegetable & legume, prepared with stock or water, homemade from basic ingredients |
| 21101012 | Soup, ham & vegetable, prepared with stock or water, homemade from basic ingredients |
| 21101020 | Soup, pork & vegetable, prepared with stock or water, homemade from basic ingredients |
| 21101025 | Soup, wonton, clear broth with dumplings |
| 21102002 | Soup, cream of vegetable, prepared with cream or milk, homemade from basic ingredients |
| 21102006 | Soup, miso, with tofu & seaweed |
| 21102007 | Soup, mixed vegetable, prepared with stock or water, homemade from basic ingredients |
| 21102008 | Soup, mixed vegetable & legume, with pasta or grains, prepared with stock or water, homemade from basic ingredients |
| 21102009 | Soup, mixed vegetable & pasta or grains, prepared with stock or water, homemade from basic ingredients |
| 21102010 | Soup, mixed vegetable & legume, prepared with stock or water, homemade from basic ingredients |
| 21102011 | Soup, potato, prepared with stock or water, homemade from basic ingredients |
| 21102014 | Soup, pumpkin, prepared with stock or water, homemade from basic ingredients |
| 21102015 | Soup, pumpkin, prepared with stock or water & coconut milk, homemade from basic ingredients |
| 21102017 | Soup, tomato, prepared with stock or water, homemade from basic ingredients |
| 21501002 | Soup, chicken & sweetcorn, ready to eat, canned |
| 21502002 | Soup, cream of vegetable, ready to eat, canned |
| 21502006 | Soup, tomato, ready to eat, canned |
| 21602002 | Soup, mixed vegetable, from cafe or restaurant |
| 23103008 | Sauce, cranberry, commercial |
| 24103005 | Potato, pale skin, peeled or unpeeled, mashed with cows milk & butter or dairy blend |
| 24103008 | Potato, peeled or unpeeled, mashed with cows milk & butter or dairy blend |
| 24103009 | Potato, peeled or unpeeled, mashed with cows milk & margarine spread |
| 24103011 | Potato, peeled, mashed, prepared, from cafe or restaurant |
| 24103012 | Potato bake, made with bacon, cheese &/or cream |
| 24103013 | Potato bake, made with cheese |
| 24901008 | Curry, homemade, potato, dairy based sauce |
| 24901009 | Curry, commercial, spinach & cheese (palak paneer) |
| 24901010 | Curry, commercial, vegetable, cream based sauce |
| 24901012 | Curry, homemade, vegetable, homemade sauce |
| 24901013 | Curry, homemade, vegetable, homemade coconut milk sauce |
| 24901017 | Curry, homemade, vegetable & legume, homemade sauce |
| 24901018 | Stir-fry, homemade, egg & vegetable |
| 24901019 | Stir-fry, homemade, egg & vegetable, with rice or noodles |
| 24901020 | Stir-fry, homemade, green vegetable, homemade sauce |
| 24901022 | Stir-fry, homemade, vegetable |
| 24901023 | Stir-fry, homemade, vegetable, with noodles |
| 24902003 | Capsicum, stuffed with meat & rice |
| 24902006 | Mushroom, stuffed with cheese & bacon |
| 24904003 | Salad, carrot, no added dressing |
| 24904004 | Salad, coleslaw, commercial, added dressing |
| 24904007 | Salad, cucumber, no added dressing |
| 24904008 | Salad, fennel, no added dressing |
| 24904023 | Salad, greek, no added dressing |
| 24904026 | Salad, potato, commercial, added dressing |
| 24904027 | Salad, potato, homemade from basic ingredients, added dressing |
| 24904028 | Salad, roast vegetable, no added dressing |
| 24904030 | Salad, tabouleh, commercial |
| 24904031 | Salad, tabouleh, homemade from basic ingredients |
| 24904032 | Salad, not further defined |
| 24905002 | Salad, Asian style, made with cabbage, carrot, onion, fresh herbs, peanuts & poached chicken, no added dressing |
| 24905003 | Salad, Asian style, made with cabbage, carrot, onion, fresh herbs, peanuts & poached chicken, with dressing |
| 24905023 | Salad, Thai beef, made with lettuce, onion, cucumber, herbs, chilli & beef, with dressing |
| 25201003 | Miso, soyabean paste |
| 25201004 | Baked beans, canned in BBQ sauce, regular |
| 25201005 | Baked beans, canned in tomato sauce, regular |
| 25201007 | Baked beans, canned in tomato & cheese sauce, regular |
| 25202001 | Bean paste |
| 25202007 | Curry, homemade, legume (dhal) |
| 25202008 | Curry or casserole, homemade, chick pea, homemade sauce |
| 25202009 | Falafel, chickpea patty, deep fried, fat not further defined |
| 25202010 | Rissole or patty, vegetable or lentil, commercial, baked, fried, grilled or BBQ'd, no added fat |
| 25202011 | Rissole or patty, vegetable or lentil, commercial, baked, fried, grilled or BBQ'd, fat not further defined |
| 25202013 | Rissole or patty, vegetable or lentil, homemade, baked, fried, grilled or BBQ'd, fat not further defined |
| 25202014 | Salad, chickpea, with vegetables |
| 25202016 | Salad, three bean, homemade from basic ingredients |

*Note.* This table reports all the recorded disaggregated dishes from the ASA24 in the MAST dataset.

# Supplementary Material B:

## Scoring method MeDi

To compute the MeDi score using the various assessment tools, we followed seven steps outlined in Figure 1: (1) relevant items from the ASA24, CCV FFQ, and EPIC FFQ were selected (included items for each assessment tool are presented in Table 3), (2) the daily grams consumed for the selected items extracted, (3) the daily serving size was determined, guided by Martinez-Gonzalez et al. (2012) (presented in Table 2 and 3), (4) the consumed servings per day were calculated for each item, (5) all items per component were weighted and summed to calculate a single value per food component, and (6) the recommended daily servings which were assigned to a component score (Table 2) were used to assign a score of 1 or 0 for each component. For example, the MeDi recommends 2 or more servings of vegetables per day. Thus, 1 point was assigned to participants that consumed 2 or more servings and 0 points were assigned to those with a vegetable intake lower than 2 servings per day. Dietary food components which are presumed to be beneficial were assigned a value of 1 if the participant consumed volumes higher or equal to the cut-off point. Reverse scoring was applied for food components that are presumed to be unhealthy (See Table 2). Lastly, (7) the dichotomous (e.g., 0 or 1) MeDi scores for each food component were summed to compute the total MeDi score for each participant. The workflow also encompasses several steps where subjective choices from the researcher are required.

## Scoring method DASH

The DASH dietary pattern score was computed in seven stages as outlined in Figure 1: (1) relevant items were selected from the ASA24, CCV FFQ and EPIC FFQ (an overview of the included items is presented in Table 4), (2) the daily grams consumed for the selected items extracted, (3) the daily serving size for each item were determined guided by Folsom et al. (2007) (as presented in Table 2 and 4). For the items where the serving size was not provided by Folsom et al. (2007), the serving size was extracted from the United States Department of Agriculture (USDA) National Nutrient Database for Standard Reference dietary guidelines (2015-2020), as the original DASH dietary pattern was scored in US serving sizes. The serving size for alcohol was extracted from the NIAA website: <https://www.niaaa.nih.gov/alcohols-effects-health/overview-alcohol-consumption/what-standard-drink>, (4) the consumed servings per day were calculated for each item, (5) all items per component were weighted and summed to calculate a single value for each food component, and (6) the recommended daily servings which were assigned to a component score provided by Folsom et al. (2007) were used to assign a full (1), a half (0.5) of no (0) points for each component (as presented in Table 2). Finally, (7) these scores were summed across the 11 components to calculate the total DASH dietary pattern score for each participant. For example, the DASH diet recommended 2 or more servings of whole grains per day. Thus, 1 point was assigned to participants that consumed 2 or more servings daily, 0.5 points were assigned to those with a whole grain intake that approached the recommended amount (> = 1 to < 2 servings/day), and 0 points were given to participants that consumed far less than the recommended amount (<1 servings/day). For the component where less intake is recommended, reverse scoring was applied. Lastly, (5) the DASH diet scores (0, 0.5 or 1) for each food component were summed to compute the DASH diet score for each participant.

## Scoring method MIND

The MIND score was computed in seven stages as outlined in Figure 1: (1) relevant items from the ASA24, CCV FFQ and EPIC FFQ were selected (as presented in Table 5), (2) the daily grams consumed for the selected items extracted, (3) the daily serving size per day for each item was determined (as presented in Table 2 and 5), (4) the consumed servings per day were calculated for each item, (5) all items per component were weighted and summed to calculate a single value for each food component. The MIND consists of 15 components including 10 healthy food components (whole grains, green leafy vegetables, other vegetables, berries, fish, poultry, beans, nuts, olive oil, and wine) and 5 unhealthy food components (red meats, fast/fried food, butter/margarine, cheese, pastries/sweets). Most of the components inquired on servings consumed per day except for olive oil (primary oil used), fast-fried foods (times per day), and wine (glass per day). (6) the recommended daily servings which were assigned to a component score provided by Morris et al. (2015) were used to assign full (1), half (0.5), or zero (0) points for each component (as presented in Table 2). For example, the MIND recommended 3 or more servings of whole grains per day. Thus, 1 point was assigned to participants that consumed 3 or more servings daily, 0.5 points were assigned to those with a whole grain intake that approached the recommended amount (> = 1 to < 3 servings/day), and 0 points were given to participants that consumed far less than the recommended amount (<1 serving/day). For the components where less intake is recommended, reverse scoring was applied. Finally, (7) these scores were summed across the 11 components to calculate the total MIND dietary pattern score for each participant.

# Supplementary Material C:

## Measures

### Biochemical Markers and Cardiovascular measures

The main objective of the supporting analysis was to compare the MeDi, DASH, and MIND tertile diet groups across demographic variables, biochemical markers, and cardiovascular indicators with the intention of assessing whether the measured variables align with the expected outcomes for each tertile group.

### Biochemical Markers

Participants were instructed to fast for a 10-hour period prior to their initial baseline testing visit. Skilled healthcare professionals such as licensed nurses or qualified venepuncture technicians, performed the blood collection procedure. During this baseline visit of the MAST trial, various biochemical markers associated with inflammation, such as high-sensitivity C-reactive protein were measured. Additionally, levels of total and high-density lipoprotein cholesterol, homocysteine, vitamin B12, vitamin B6, folate, and blood glucose. In the PLICAR trial, only vitamin B12, vitamin B6, red blood cell folate, and homocysteine blood levels were measured, while the CANN trial focused on high-sensitivity C-reactive protein, total cholesterol, high-density lipoprotein-cholesterol, and glucose. The measure of fasting blood glucose provides an understanding of the participant's glucoregulatory efficiency and control. The samples were then sent to either a commercial laboratory or analysed on-site.

### Cardiovascular measures

#### Blood Pressure and Heart Rate

To measure the brachial blood pressure and heart rate, a non-invasive SphygmoCor XCEL device or a sphygmomanometer was utilized. Following a five-minute rest period, while participants assumed a supine position and a blood pressure cuff was placed around their arm (either right or left), three measurements were taken.

#### Pulse Wave Velocity

Pulse wave velocity (PWV), which is a marker of arterial stiffness, was assessed using an appropriately sized femoral cuff. In the MAST, PLICAR, and CANN-Melbourne site trials, the SpygmoCor XCEL was used, while the Vicorder was used in the CANN-UK site trial. These devices recorded the blood pressure pulse waveform at the femoral artery level. Simultaneously, a small, pressure-sensitive probe was placed on the participant's neck to obtain the blood pressure pulse waveform at the carotid artery. By measuring the distance between the neck measurement point and the leg cuff, the velocity of the pressure pulse through the large arteries was measured.

## Supporting Analysis

All statistical analyses were conducted using IBM SPSS Statistics for Mac version 29 (IBM Corp., Armonk, NY, USA). The dietary patterns (MeDi, DASH, and MIND) were modelled as non-linear associations, specifically categorized into tertiles. The determination of the cut-off points of their tertiles was based on a data-driven approach, with each tertile representing approximately 33.3% of the participants. Table S6 to S9 present the analysis of literature-based tertiles and quintiles as reported in the original papers, aiming to elucidate any cohort differences between our dataset and the datasets of the original papers.

Before conducting the analysis, the variables were screened for normality and outliers. In addition, Energy over- and under-reporting was assessed, and due to unplausible energy intake (>5000 kcal/day) one participant from the MAST dataset was excluded from the analysis After this exclusion, the analysis consisted of 140 participants for the MAST trial, 196 for PLICAR, 145 for the CANN UK site, and 101 for the CANN Australia site analysis. Further, the occurrence of covariates was assessed through ANOVA for continuous variables and chi-square analysis for categorical variables. Descriptive statistics, including mean and standard deviations and percentages, were utilized to report the demographic characteristics. An analysis of covariance (ANCOVA) was employed to compare biomarkers of nutrient status and cardiovascular measures across the tertile groups of the dietary patterns (MeDi, DASH, MIND). The statistical significance level was set at p < 0.05.

### Covariates

Regarding the covariates, the analysis revealed significant differences in various characteristics between the terilte groups of the MeDi, DASH and MIND in different datasets. For example, in the MAST dataset, both body mass index (BMI) and sex demonstrated significant differences among the MIND diet groups, while BMI significantly varied among the MeDi groups. In the PLICAR dataset, significant differences in years of education were observed between the DASH diet groups, and sex differed significantly among the MIND diet groups. Furthermore, the CANN-UK and CANN- Melbourne datasets indicated significant differences in gender and years of education among the MIND diet groups. To ensure consistency throughout the manuscript, all analyses were controlled for years of education, BMI, and sex (1). In addition, the analysis was controlled for age as some biochemical levels and cardiovascular functions tend to differ in middle to older individuals (2).

## Supporting Results

### Sample Characteristics

The sample descriptions of the three data sources are presented in Table S2. According to the MAST trial, those who adhered closely to the MIND or MeDi diet tended to have a lower BMI. Additionally, there was a higher proportion of females among those who adhere to the MIND. In the PLICAR trial, female participants also showed the highest adherence to the MIND diet. Meanwhile, individuals who adhered to the DASH diet had predominantly a longer education. In the CANN trials, females showed a greater tendency to adhere to the MIND dietary patterns compared to males in both study sites. Additionally, those who adhered closely to the MIND had a longer overall education. No other significant demographic differences were observed in the MAST, PLICAR, CANN-UK and CANN- Melbourne trials.

| Table S2: Characterization of analysed MAST, PLICAR, CANN-Melbourne, CANN-UK participants according to data driven tertiles of the MeDi, DASH and MIND diet score. | | | | | | | | | | | | | | | | | | | | | | | | | | | | |
| --- | --- | --- | --- | --- | --- | --- | --- | --- | --- | --- | --- | --- | --- | --- | --- | --- | --- | --- | --- | --- | --- | --- | --- | --- | --- | --- | --- | --- |
|  |  | **All** |  |  |  | **MeDi** | | | | | | |  | **DASH** | | | | | | |  | **MIND** | | | | | |  |
|  |  |  |  |  |  | **lowest tertile** | | **Second tertile** | | **Higherst tertile** | | |  | **lowest tertile** | | **Second tertile** | | **Higherst tertile** | |  |  | **lowest tertile** | | **Second tertile** | | **Higherst tertile** | |  |
| **MAST** |  | **n** | **Mean** | **SD** |  | **Mean** | **SD** | **Mean** | **SD** | **Mean** | **SD** | **p** |  | **Mean** | **SD** | **Mean** | **SD** | **Mean** | **SD** | **p** |  | **Mean** | **SD** | **Mean** | **SD** | **Mean** | **SD** | **p** |
| **Age** |  | 140 | 53.28 | 0.65 |  | 52.51 | 6.70 | 56 | 6.43 | 52 | 7.12 | 0.06 |  | 51.89 | 6.57 | 52.84 | 6.82 | 53.96 | 7.26 | 0.10 |  | 52.47 | 6.66 | 53.39 | 6.98 | 52.82 | 7.08 | 0.80 |
| **Education (years)** |  | 140 | 17.07 | 0.32 |  | 16.72 | 3.59 | 17 | 3.08 | 17 | 3.05 | 0.71 |  | 16.63 | 3.50 | 16.89 | 3.50 | 17.36 | 3.07 | 0.24 |  | 16.44 | 3.78 | 16.74 | 3.02 | 17.69 | 3.20 | 0.19 |
| **BMI** |  | 140 | 26.96 | 0.50 |  | 27.98 | 5.15 | 27 | 4.61 | 25 | 5.28 | 0.02* |  | 28.61 | 6.00 | 26.25 | 4.06 | 26.77 | 5.10 | 0.17 |  | 28.20 | 4.61 | 28.48 | 5.83 | 24.88 | 4.31 | 0.001* |
| ***Gender (n, %)*** |  |  |  |  |  |  |  |  |  |  |  | 0.25 |  |  |  |  |  |  |  | 0.30 |  |  |  |  |  |  |  | 0.009* |
| **Femal** |  | 71 |  | 50.00 |  | 37 | 45.70 | 11 | 50.00 | 23 | 62.20 |  |  | 34 | 75.63 | 19 | 42.22 | 18 | 50.00 |  |  | 16 | 33.30 | 28 | 56.00 | 27 | 64.30 |  |
| **Male** |  | 69 |  | 49.30 |  | 44 | 54.30 | 11 | 50.00 | 14 | 37.80 |  |  | 25 | 42.40 | 26 | 57.80 | 18 | 50.00 |  |  | 32 | 66.70 | 22 | 44.00 | 15 | 35.70 |  |
| ***Ethnicity (n,%)*** |  |  |  |  |  |  |  |  |  |  |  | 0.10 |  |  |  |  |  |  |  | 0.43 |  |  |  |  |  |  |  | 0.12 |
| **Caucasian** |  | 107 |  | 76.10 |  | 66 | 81.50 | 14 | 63.64 | 28 | 75.68 |  |  | 43 | 72.88 | 35 | 77.80 | 30 | 83.33 |  |  | 41 | 85.42 | 38 | 76.00 | 29 | 69.05 |  |
| **Asian** |  | 10 |  | 7.00 |  | 2 | 2.50 | 3 | 13.64 | 5 | 13.51 |  |  | 3 | 5.10 | 4 | 8.89 | 3 | 8.33 |  |  | 0 | 0.00 | 4 | 8.00 | 6 | 14.29 |  |
| **Other** |  | 23 |  | 16.20 |  | 13 | 16.00 | 5 | 22.73 | 4 | 10.81 |  |  | 13 | 22.03 | 6 | 13.33 | 3 | 8.33 |  |  | 7 | 14.58 | 8 | 16.00 | 7 | 16.67 |  |
|  |  |  |  |  |  |  |  |  |  |  |  |  |  |  |  |  |  |  |  |  |  |  |  |  |  |  |  |  |
| **PLICAR** |  |  |  |  |  |  |  |  |  |  |  |  |  |  |  |  |  |  |  |  |  |  |  |  |  |  |  |  |
| **Age** |  | 164 | 65.5 | 6.29 |  | 66.2 | 6.91 | 64.4 | 5.04 | 65.4 | 6.44 | 0.39 |  | 65.2 | 6.32 | 67.5 | 6.496 | 64.6 | 6.3 | 0.14 |  | 66.5 | 7.05 | 65.7 | 5.47 | 64.42 | 6.3636 | 0.23 |
| **Education (years)** |  | 192 | 15.1 | 3.4 |  | 14.9 | 3.1 | 14.8 | 3.81 | 15.7 | 3.55 | 0.37 |  | 14.8 | 3.47 | 14.6 | 3.371 | 16.2 | 3.12 | 0.037* |  | 15.1 | 3.34 | 15.2 | 3.06 | 15.04 | 3.7988 | 0.97 |
| **BMI** |  | 178 | 28.1 | 4.94 |  | 28.2 | 4.52 | 27.7 | 6.1 | 28.1 | 5.05 | 0.87 |  | 29 | 5.33 | 27.9 | 5.1 | 26.8 | 4.21 | 0.09 |  | 28.2 | 4.38 | 28.6 | 5.65 | 27.35 | 5.1667 | 0.41 |
| ***Gender (n, %)*** |  |  |  |  |  |  |  |  |  |  |  | 0.69 |  |  |  |  |  |  |  | 0.41 |  |  |  |  |  |  |  | <0.001* |
| **Femal** |  | 106 |  | 54.4 |  | 46 | 52.3 | 30 | 55.6 | 23 | 60.5 |  |  | 46 | 59 | 29 | 56.9 | 24 | 47.1 |  |  | 30 | 44.1 | 25 | 45.5 | 44 | 77.2 |  |
| **Male** |  | 89 |  | 45.6 |  | 42 | 47.7 | 24 | 44.4 | 15 | 39.5 |  |  | 32 | 41 | 22 | 43.1 | 27 | 52.9 |  |  | 38 | 55.9 | 30 | 54.5 | 13 | 22.8 |  |
| ***Ethnicity (n,%)*** |  |  |  |  |  |  |  |  |  |  |  | 0.06 |  |  |  |  |  |  |  | 0.52 |  |  |  |  |  |  |  | 0.75 |
| **Caucasian** |  | 165 |  | 84.6 |  | 75 | 85.2 | 47 | 87 | 33 | 86.8 |  |  | 66 | 84.6 | 44 | 86.3 | 45 | 88.2 |  |  | 57 | 83.8 | 50 | 90.9 | 48 | 84.2 |  |
| **Asian** |  | 9 |  | 4.6 |  | 2 | 2.3 | 5 | 9.3 | na | na |  |  | 4 | 5.1 | 3 | 5.9 | 0.00 | na |  |  | 3 | 4.4 | 2 | 3.6 | 2 | 3.5 |  |
| **Other** |  | 21 |  | 10.8 |  | 11 | 12.5 | 2 | 3.7 | 5 | 13.2 |  |  | 8 | 10.3 | 4 | 7.8 | 6 | 11.8 |  |  | 8 | 11.8 | 3 | 5.5 | 7 | 12.3 |  |
|  |  |  |  |  |  |  |  |  |  |  |  |  |  |  |  |  |  |  |  |  |  |  |  |  |  |  |  |  |
| **CANN - SUT** |  |  |  |  |  |  |  |  |  |  |  |  |  |  |  |  |  |  |  |  |  |  |  |  |  |  |  |  |
| **Age** |  | 101 | 65.14 | 6.57 |  | 64.8 | 6.61 | 66.1 | 6.29 | 64.9 | 7.22 | 0.70 |  | 64.83 | 6.42 | 66.75 | 7.38 | 64.06 | 5.72 | 0.26 |  | 64.95 | 7.67 | 65.97 | 6.10 | 64.26 | 5.55 | 0.60 |
| **Education (years)** |  | 101 | 15.25 | 3.93 |  | 15 | 3.42 | 14.5 | 4.4 | 16.8 | 4.42 | 0.13 |  | 14.46 | 3.05 | 14.94 | 3.61 | 16.45 | 5.09 | 0.12 |  | 14.89 | 3.26 | 14.37 | 4.25 | 17.38 | 3.90 | 0.014* |
| **BMI** |  | 98 | 27.79 | 4.82 |  | 28.1 | 4.67 | 27.1 | 4.42 | 27.7 | 5.8 | 0.66 |  | 28.68 | 4.54 | 26.55 | 5.11 | 27.58 | 4.60 | 0.19 |  | 29.01 | 4.90 | 27.40 | 4.61 | 25.92 | 4.45 | 0.06 |
| ***Gender (n, %)*** |  |  |  |  |  |  |  |  |  |  |  | 0.10 |  |  |  |  |  |  |  | 0.71 |  |  |  |  |  |  |  | 0.006* |
| **Femal** |  | 63 |  | 62.4 |  | 29 | 58 | 16 | 53.3 | 15 | 83.3 |  |  | 24 | 64.9 | 20 | 62.5 | 13 | 44.8 |  |  | 15 | 41.7 | 28 | 68.3 | 17 | 81 |  |
| **Male** |  | 38 |  | 37.6 |  | 21 | 42 | 14 | 46.7 | 3 | 16.7 |  |  | 13 | 35.1 | 12 | 37.5 | 16 | 55.2 |  |  | 21 | 58.3 | 13 | 31.7 | 4 | 19 |  |
|  |  |  |  |  |  |  |  |  |  |  |  |  |  |  |  |  |  |  |  |  |  |  |  |  |  |  |  |  |
| **CANN - UK** |  |  |  |  |  |  |  |  |  |  |  |  |  |  |  |  |  |  |  |  |  |  |  |  |  |  |  |  |
| **Age** |  | 145 | 65.7 | 6.41 |  | 66.10 | 6.51 | 65.55 | 6.05 | 65.21 | 6.59 | 0.79 |  | 65.75 | 6.59 | 65.06 | 5.78 | 66.51 | 6.63 | 0.61 |  | 66.29 | 5.60 | 65.67 | 7.14 | 65.32 | 6.38 | 0.76 |
| **Education (years)** |  | 145 | 14 | 3.5 |  | 14.04 | 3.89 | 13.94 | 2.99 | 14.07 | 2.91 | 0.99 |  | 13.90 | 3.90 | 13.97 | 3.02 | 14.27 | 3.28 | 0.87 |  | 13.78 | 3.37 | 14.64 | 3.99 | 13.45 | 2.85 | 0.021* |
| **BMI** |  | 145 | 26.2 | 3.82 |  | 26.50 | 4.10 | 26.14 | 3.03 | 25.61 | 3.82 | 0.56 |  | 26.55 | 3.95 | 25.81 | 3.58 | 26.09 | 3.88 | 0.63 |  | 26.39 | 4.23 | 26.43 | 3.68 | 25.76 | 3.53 | 0.67 |
| ***Gender (n, %)*** |  |  |  |  |  |  |  |  |  |  |  | 0.14 |  |  |  |  |  |  |  | 0.782 |  |  |  |  |  |  |  | 0.003* |
| **Femal** |  | 79 |  | 51.7 |  | 40 | 47.6 | 16 | 51.6 | 20 | 69 |  |  | 34 | 50 | 20 | 57.1 | 22 | 53.7 |  |  | 18 | 35.3 | 31 | 56.4 | 27 | 71.1 |  |
| **Male** |  | 69 |  | 46.9 |  | 44 | 52.4 | 15 | 48.4 | 9 | 31 |  |  | 34 | 50 | 15 | 42.9 | 19 | 46.3 |  |  | 33 | 64.7 | 24 | 43.6 | 11 | 28.9 |  |
| Comparison by tertile groups was performed using ANOVA for continuous variables, and chi-square tests for categorical variables. BMI = Body mass index, DASH = Dietary Approach to Systolic Hypertension diet, MeDi = Mediterranean diet, MIND = Mediterranean-DASH diet intervention for neurodegenerative delay diet, n = number of participants, SD = standard deviation, UK = United Kingdom. * p < 0.05. | | | | | | | | | | | | | | | | | | | | | | | | | | | | |

### Biochemical Markers and Cardiovascular Outcomes

The analysis includes both the use of data-driven and literature-based cut-off point methods. The analysis was adjusted for years of education, BMI, gender and age. The data-driven cut-off points method observed significant differences such as reduced diastolic blood pressure (p = 0.039) and pulse wave velocity measures (p = 0.003), a marker of arterial stiffness for high MeDi adherence in the MAST trial (Table S3). However, despite most of the measures changing as expected, there were no other significant differences present across the dietary pattern tertile groups, biochemical markers and cardiovascular measures. In addition, Gauci et al. (2022) reported a correlation between the continuous variables of the scored dietary patterns derived from the MAST trial and self-reported diet quality, thereby providing additional validation for our dietary pattern scoring methods (3).

As presented in Tables S4 to S6, no significant differences were shown across the dietary pattern tertile groups in the PLICAR, CANN-Melbourne and CANN-UK datasets. All analyses were adjusted for years of education, BMI, gender, and age.

Tables S7 to S10 present literature-based cut-off points, showcasing the notable distinctions between our study populations and those included in the original studies (Martinez-Gonzalez et al. (2012), Folsom et al. (2007) and Morris et al. (2015)). Particularly, when applying the literature-based cut-off points for the Mediterranean diet, a majority of participants were observed to fall within the first tertile group, with only one, two, or zero participants in the second and third tertile groups. Similarly, the literature-based cut-off points for DASH ranged from 0.5 to 10, which wasn’t all-inclusive for our population, as the score ranged from 0 to 7.5. These observations highlight the limitations of using literature-based cut-off points in analyses. However, using literature-based cut-off points when feasible, can facilitate cross-study comparisons of results.

It's worth noting that using literature-based cut-off points eliminated the significant differences in our findings apart from individuals in the highest tertile group of the MIND diet having significantly lower high-sensitivity C-reactive protein, a marker for inflammation (p = 0.045) compared to the second tertile group in the CANN-Melbourne trial.

##### Data-Driven Tertiles Tables

##### Literature based Tertiles Tables

1. Gauci S, Young LM, Arnoldy L, Lassemillante A-C, Scholey A, Pipingas A. Dietary patterns in middle age: effects on concurrent neurocognition and risk of age-related cognitive decline. Nutrition Reviews. 2022;80(5):1129-59.

2. Fleg JL, Strait J. Age-associated changes in cardiovascular structure and function: a fertile milieu for future disease. Heart failure reviews. 2012;17:545-54.

3. Gauci S, Young LM, Arnoldy L, Scholey A, White DJ, Lassemillante A-C, et al. The Association Between Diet and Cardio-Metabolic Risk on Cognitive Performance: A Cross-Sectional Study of Middle-Aged Australian Adults. Frontiers in Nutrition. 2022;9.
